# Accuracy of electrical impedance tomography to detect perfusion defects in pulmonary embolism

**DOI:** 10.1186/s13054-026-06073-y

**Published:** 2026-05-20

**Authors:** Eduardo Augusto Pinto Rodrigues, Eder Chaves Pacheco, Maria Aparecida Miyuki Nakamura, Ana Carolina Cardoso dos Santos, Jade Lara de Melo, Glauco M. Plens, Glasiele Cristina Alcala, Marcus Victor, Yi Xin, Maurizio Cereda, Susimeire Gomes, Hye Ju Lee, Bruno M. Ribeiro, Rafael M. Ianotti, Orival Freitas-Filho, Jose Leonidas Alves-Jr, Lorenzo Berra, Caio C. A. Morais, Larissa Bertacchini, Mikuláš Mlček, Rogerio Souza, João Batista Borges, Eduardo L. V. Costa, Marcelo B. P. Amato

**Affiliations:** 1 Laboratorio de Pneumologia LIM‑09, Faculdade de Medicina, Hospital das Clinicas HCFMUSP, Universidade de Sao Paulo, Sao Paulo, Brazil SP; 2 Divisao de Pneumologia, Faculdade de Medicina, Instituto do Coracao, Hospital das Clinicas HCFMUSP, Universidade de Sao Paulo, Sao Paulo, SP Brazil; 3 Department of Anesthesia, Critical Care, and Pain Medicine, Massachusetts General Hospital, Boston, MA United States of America; 4 Department of Anesthesia, Critical Care, and Pain Medicine, Harvard Medical School, Boston, MA United States of America; 5 Department of Radiology, Hospital das Clinicas da Faculdade de Medicina da Universidade de Sao Paulo, Sao Paulo, SP Brazil; 6 Heart Institute (InCor), Hospital das Clinicas, Faculdade de Medicina da Universidade de Sao Paulo, SP Sao Paulo, Brazil; 7 Cardio-Pulmonary Department, Cardiovascular and Thoracic Surgery Division, Heart Institute (InCor), Hospital das Clinicas, Faculdade de Medicina da Universidade de Sao Paulo, Sao Paulo, Brazil; 8 Cardio-Pulmonary Department, Interventional Cardiology Division, Heart Institute (InCor), Hospital das Clinicas, Faculdade de Medicina da Universidade de Sao Paulo, Sao Paulo, Brazil; 9 Unidade de Terapia Intensiva Adulto, Hospital das Clínicas da Universidade Federal de Pernambuco, Recife, Brazil; 10 Institute of Physiology, 1st Faculty of Medicine, Charles University, Prague, Czech Republic; 11 National Medical Institute of the Ministry of Interior and Administration, Warsaw, Poland

**Keywords:** Bedside monitoring, Critical care, Electrical impedance tomography, Pulmonary embolism, Pulmonary perfusion, Ventilation–perfusion mismatch

## Abstract

**Background:**

Timely detection of pulmonary embolism (PE) is crucial, particularly in critical settings where rapid confirmation or exclusion is required and computerized tomography pulmonary angiography (CTPA) poses safety challenges. We assessed the experimental and clinical accuracy of electrical impedance tomography (EIT)-ventilation-perfusion (V̇/Q̇) maps and a novel *wasted-ventilation* index for detecting PE.

**Methods:**

Ten piglets underwent EIT–V̇/Q̇ mapping before and after proximal or distal pulmonary artery occlusions. EIT-perfusion maps were validated against dynamic contrast-enhanced CT (DCE-CT) and quantitative *clot-burden* analysis from whole-lung CTPA. To assess specificity, models of non-occlusive perfusion impairment were added (6 piglets). The *wasted-ventilation* index was refined across 114 piglet conditions (66 PE) and subsequently validated in 66 patients with acute respiratory failure (257 exams) and 10 patients with chronic thromboembolic disease (31 exams), totaling 288 EIT-exams.

**Results:**

Strong positive correlations between estimates of regional perfusion obtained by EIT vs. DCE-CT or CTPA were found. Agreement showed mean bias of − 3.04 ± 3.02% between EIT and DCE-CT, and + 3.45 ± 2.81% between EIT and CTPA. The *wasted-ventilation* index performed with AUC = 0.989 in animals (*P* < 0.0001; sensitivity 96%, specificity 94%) and AUC = 0.923 in patients (*P* < 0.001; sensitivity 81%, specificity 94%). The index consistently decreased after thrombolysis.

**Conclusions:**

The novel *wasted-ventilation* index showed high accuracy in detecting pulmonary arterial occlusions of varying severity, distinguishing them from other perfusion problems. Its agreement with CTPA, DCE-CT, and reference methods supports EIT–V̇/Q̇ mapping as a reliable diagnostic aid when conventional imaging is unfeasible or risky.

**Supplementary Information:**

The online version contains supplementary material available at 10.1186/s13054-026-06073-y.

## Background

Ventilation–perfusion (V̇/Q̇) mismatch is a defining feature of acute respiratory failure and the leading mechanism underlying blood-gas derangement across a wide range of clinical conditions [1]. Various therapeutic interventions improve gas exchange primarily by reducing this mismatch [2]. Pulmonary embolism (PE), a prototypical disorder of V̇/Q̇ imbalance, is both common and potentially fatal, with an estimated mortality rate of 32.3 per 100,000 population in the United States [3]. Early diagnosis is critical for improving outcomes [4], yet confirmation may be challenging when computed tomography pulmonary angiography (CTPA) is contraindicated, unfeasible, or unavailable [5]. Although several bedside techniques, such as echocardiography, have been investigated for PE detection, they are limited by low sensitivity and a high degree of operator dependence.

Electrical impedance tomography (EIT) is a non-invasive, radiation-free imaging modality that provides real-time bedside monitoring of lung function [6]. Changes in thoracic impedance reflect cyclic variations in pulmonary air and blood content, enabling continuous assessment of both ventilation [7] and perfusion [8–11]. By integrating these signals, EIT can estimate V̇/Q̇ mismatch [9, 11, 12, 13, 14] and track dynamic changes induced by therapeutic interventions [11] such as prone positioning [15], inhaled nitric oxide [10], or recruitment maneuvers [13].

The present study aimed to determine the diagnostic accuracy of EIT-derived V̇/Q̇ maps for detecting perfusion defects produced by experimentally induced PE and for distinguishing them from non-occlusive perfusion impairments. First, EIT perfusion maps were validated against dynamic contrast-enhanced CT (DCE-CT). Second, their agreement with the quantitative *clot-burden* derived from whole-lung CTPA, independently assessed by a blinded radiologist, was evaluated. The validated EIT perfusion maps were then integrated with ventilation maps to generate EIT-based V̇/Q̇ distributions and a derived *wasted-ventilation* index. The diagnostic performance of these measures was analyzed across a spectrum of vascular occlusion intensities and under experimental conditions of perfusion impairment not related to vascular obstruction but caused by matched ventilation defects. Finally, clinical validation was conducted in hypoxemic patients with and without PE (either acute or chronic), including paired EIT recordings before and after thrombolysis or surgical removal.

## Methods

Full methods are provided in the Supplementary Material.

### Experimental Data

The study was approved by the local Ethics Committee. Ten healthy piglets were ventilated, anesthetized, paralyzed, and monitored as described elsewhere [16].

Experimental Outline.

Perfusion assessments were acquired using EIT, DCE-CT, and CTPA at baseline, after inducing a proximal pulmonary artery occlusion by inflating the balloon of the pulmonary artery catheter at a proximal level, and after inducing a distal pulmonary artery occlusion by inflating the balloon at a distal level, in both lungs.

A clear transition from pulmonary artery to wedged waveforms was used as the gold standard to classify a condition as a pulmonary artery occlusion, either proximal or distal.

Bronchial obstruction was induced by selectively intubating the right or left lung and surgically sealing the trachea around the endotracheal tube. Prior bilateral ventilation with fraction of inspired oxygen (F_I_O_2_) of 0.21 for 15 min ensured high nitrogen content distal to the occluded airways, generating controlled hypoventilation and regional hypoxia while minimizing absorption atelectasis. The resulting hypoxic pulmonary vasoconstriction produced a condition of matched V̇/Q̇ impairment in the obstructed lung. Perfusion measurements by EIT and DCE-CT were obtained 15 min after obstruction.

To enrich our dataset, we further included data from another study [16]. These additional piglets (six) underwent bronchial obstruction steps, creating 3 conditions: (a) bilateral ventilation, (b) bronchial obstruction performed under F_I_O_2_ of 0.21; and (c) bronchial obstruction performed under F_I_O_2_ of 1.0. This inclusion increased the number of “challenging” control cases (cases with matched hypoperfusion and hypoventilation, but without pulmonary artery occlusion). The use of F_I_O_2_ of 1.0 during bronchial obstruction mimicked a condition of alveolar consolidation.

### Imaging studies

#### EIT

EIT continuously measured thoracic impedance variations at a rate of 50 Hz (Enlight 2100, Timpel Medical, Sao Paulo, Brazil). Extra-small electrode bands (60 mm-thick) were placed inferior to the piglet’s glenohumeral joint according to the manufacturer’s recommendation. A pneumotachograph was placed proximally to the endotracheal tube and connected to the EIT monitor. The data were then reconstructed into an image consisting of a 32 by 32-pixel spatial matrix [7]. Ventilation data were obtained by measuring tidal impedance changes (ΔZ) for each pixel, averaged over 10 breaths. ΔZ correlates well with CT measures of tidal volume [17]. EIT ΔZ will be referred to as EIT ventilation.

EIT lung perfusion was conducted in each tested condition as previously described [8]. A bolus of 10% sodium chloride solution (10 mL) was injected over one second into a central venous catheter during 20 s of apnea in continuous positive airway pressure (CPAP) mode, using the positive end-expiratory pressure (PEEP) level measured in the ventilator before the apnea. The impedance versus time waveform of each pixel was acquired and exported to a custom LabVIEW software (National Instruments, Austin, TX). Each pixel’s impedance versus time curve was analyzed for a biphasic model as previously described [8]. If two components were present, the first appearing component indicated pre-lung signal from the right heart phase or vascular tissue. The resulting lung curve was fitted to a single gamma function which was then reconstructed into a 32 by 32-pixel matrix overlapping the ventilation matrix. Using the first-pass kinetic method [8], the maximum slope of each pixel’s gamma function was calculated yielding a relative regional perfusion map.

#### EIT Post-processing

For each individual animal, EIT maps of ventilation and perfusion were normalized to obtain a maximum pixel value of 1.0 within each map. Pixels with values less than 5% of the maximum were excluded. Next, each animal’s ventilation and perfusion maps were expressed as one (global), two (right and left lung), and the following four quadrants’ regions-of-interests (ROIs): upper-right (UR), upper-left (UL), lower-right (LR), and lower-left (LL). Pixel-wise ventilation and perfusion values were summed within each ROI and divided by the sum of the global map, yielding percent values of regional ventilation and perfusion directed to each ROI.

By comparing pixel-wise ventilation and perfusion maps, we attributed to each pixel a V̇/Q̇ matching value, generating EIT V̇/Q̇ maps.

For each EIT V̇/Q̇ map, three V̇/Q̇ compartments were computed:

- *Mostly Ventilated* (EIT_HighV/Q_): Pixels with V̇/Q̇ > 2.0.
- *Ventilated and Perfused* (EIT_BalancedV̇/Q_): Pixels with V̇/Q̇ between 0.5 and 2.0.
- *Mostly Perfused* (EIT_LowV̇/Q_): Pixels with V̇/Q̇ < 0.5.

Finally, within each ROI (including the global ROI or global V̇/Q̇ map), we computed the regional index of *wasted-ventilation*: the percentage of ventilation directed to pixels classified as *Mostly Ventilated* within the ROI, in relation to the total ventilation received by that respective ROI.

#### DCE-CT

Measurements were taken during a 30-s apnea at end-expiratory pressure just above the diaphragm, with a slab thickness of 19.2 mm. A 20-mL nonionic iodinated contrast was injected into the right atrium at a rate of 10mL/s. The gas fraction was obtained at the beginning of acquisition before contrast injection. The data were measured in mL/min using the steepest slope method [18] and expressed as a percentage of total perfusion. Lung density, raw perfusion, and normalized perfusion (raw perfusion divided by lung density) were calculated for each ROI: right and left lung. The ROI where the tip of the Swan-Ganz catheter was located (or where we performed the bronchial obstruction) was called *target ROI*.

#### CTPA

**Fig. 1:**
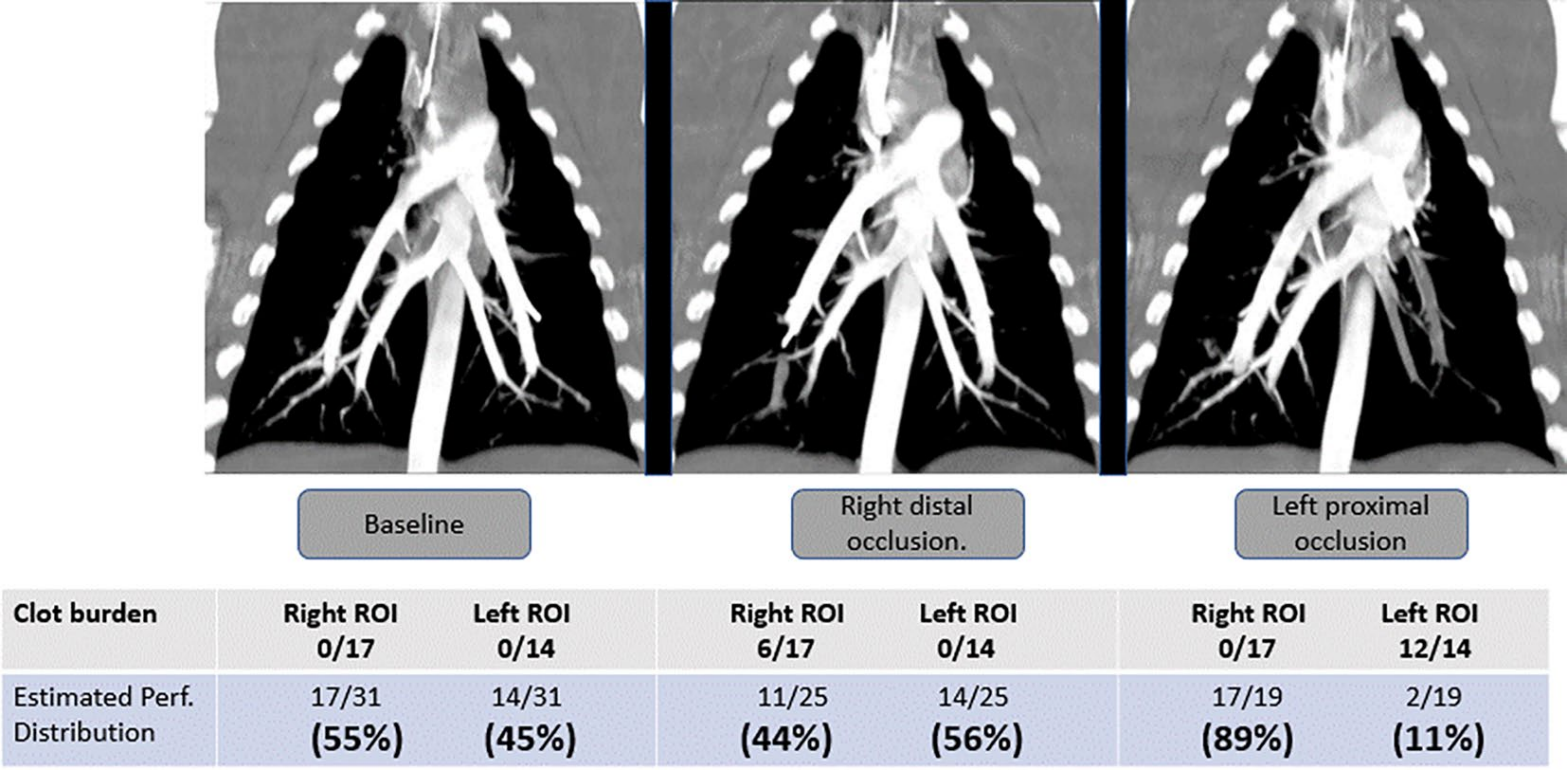
Quantification of perfusion distribution by computed tomography pulmonary angiography. This illustrative case shows how perfusion distributions are computed by tomographic pulmonary angiography (CTPA) using the *clot-burden* concept. At baseline (without any occlusion), there are 17 patent vessels in the right lung and 14 patent vessels in the left one, resulting in a perfusion distribution of 55% and 45%, respectively. According to the *clot-burden* concept, perfusion distribution is calculated based on the percentage of *patent vessels* within the region-of-interest (ROI), in relation to the whole lung, which originally presented 31 patent vessels in total. After inducing a distal pulmonary artery occlusion in the right lung (mid panel), six vessels became occluded, with 11 patent vessels remaining within the right lung, plus all 14 vessels kept patent in the left one. This resulted in 25 (31 − 6) patent vessels within the whole lung, and a perfusion distribution of 44% (11/25) for the right lung, with 56% (14/25) for the left one. After inducing proximal pulmonary artery occlusion in the left lung (right panel), 2 out of 14 vessels remained patent in the left lung, with all 17 vessels kept patent in the right lung. The whole lung has now only 19 patent vessels, and the result was 89% (17/19) perfusion directed to the right lung, and 11% (2/19) to the left lung

Iodinated contrast (1 mL/kg) at a flow rate of 8 mL/s was injected, and images 0.625 mm-thick were obtained as soon as the contrast agent reached the main pulmonary artery. CTPA data were quantified after image analysis by a cardiothoracic radiologist unaware of the EIT results. Perfusion distribution was calculated by the *clot-burden* (Fig. 1).

**Fig. 2:**
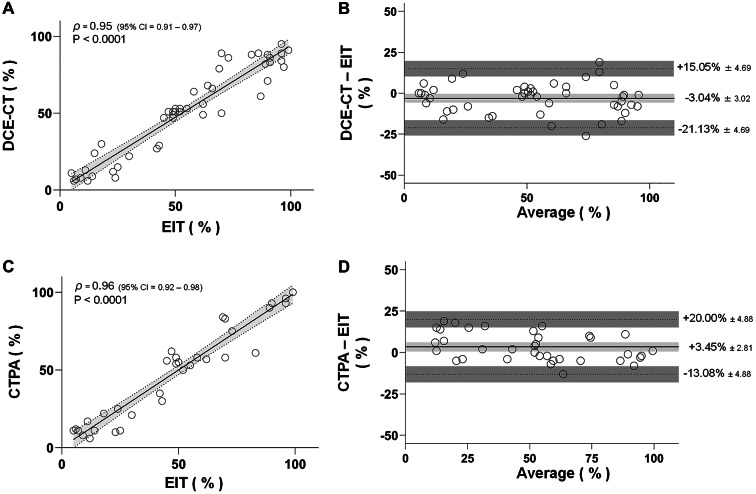
Correlations and Bland-Altman plots between dynamic contrast-enhanced computed tomography and computed tomography pulmonary angiography versus electrical impedance tomography. (**A**) Correlation between the perfusion distribution directed to the right lung region-of-interest (ROI) estimated by dynamic contrast-enhanced computed tomography (DCE-CT) and electrical impedance tomography (EIT). *ρ* = 0.95 (95% CI: 0.91 to 0.97), *P* < 0.0001. (**B**) Bland-Altman plot for the agreement between the perfusion distribution directed to the right lung ROI estimated by DCE-CT and EIT. The solid horizontal line represents the mean bias between methods, and the dashed lines indicate the upper and lower limits of agreement (LoA; mean bias ± 1.96 standard deviation). Shaded areas around the bias and around each LoA represent the corresponding 95% confidence intervals, reflecting the uncertainty in the estimation of these parameters. Mean bias ± 1.96 standard error was − 3.04 ± 3.02% (95% CI: −5.7 to −0.33%), the lower limit of agreement was − 21.13 ± 4.69% (95% CI: −25.82 to −16.43%), and the upper limit of agreement was 15.05 ± 4.69% (95% CI: 10.35 to 19.74%). (**C**) Correlation between the perfusion distribution directed to the right lung ROI estimated by computed tomography pulmonary angiography (CTPA) and EIT. *ρ* = 0.96 (95% CI: 0.92 to 0.98), *P* < 0.0001. (**D**) Bland-Altman plot for the agreement between the perfusion distribution directed to the right lung ROI estimated by CTPA and EIT. The solid horizontal line represents the mean bias between methods, and the dashed lines indicate the upper and lower LoA. Shaded areas around the bias and around each LoA represent the corresponding 95% confidence intervals, reflecting the uncertainty in the estimation of these parameters. Mean bias ± 1.96 standard error was 3.45 ± 2.81% (95% CI: 0.64 to 6.27%), a lower limit of agreement of -13.08 ± 4.88% (95% CI: -17.96 to -8.20%), and an upper limit of agreement of 20.0 ± 4.88% (95% CI: 15.12 to 24.88%)

### Patient data

Our clinical validation sample consisted of EIT-perfusion studies obtained from 66 patients with acute hypoxemic respiratory failure previously included in three different studies and 10 patients with chronic thromboembolic pulmonary hypertension (CTEPH) [19]. The approvals by the ethical committees are provided in detail in the Supplementary Material. EIT perfusion studies were pre-planned according to protocol design or indicated by the attending physician because of a high suspicion of PE (a combination of D-dimer > age-adjusted-cutoff and Geneva score > 10, found in 18 cases) and the impossibility of immediate transportation to CT or single-photon emission computed tomography (SPECT) machines.

The diagnosis of PE was confirmed in 7 of 18 patients with acute hypoxemic respiratory failure and high clinical suspicion, based on CTPA or pulmonary perfusion SPECT. An additional hypoxemic patient had PE identified incidentally on CT performed at ICU admission. For PE-negative cases, patients met one of two criteria: (a) absence of clinical suspicion with negative non-invasive rule-out tests [3] indicating low pre-test probability, or (b) non-confirmatory CTPA or perfusion mapping, regardless of pre-test probability or non-invasive assessments. The latter applied to the remaining 11 patients with hypoxemic respiratory failure and high clinical suspicion.

The CTEPH patients were studied by EIT before and after pulmonary thromboendarterectomy (PTE).

### Multiple logistic regression models and statistical analysis

We realized that the global index of *wasted-ventilation* correlated well with large *clot-burdens*. However, smaller *clot-burdens*, often producing localized clusters of EIT_HighV̇/Q_ pixels, were commonly missed. To enhance sensitivity, we calculated additional indexes of *wasted-ventilation* within each of the smaller ROIs, testing whether their inclusion in multiple logistic regression models could improve our performance in detecting PE. Each of those regional indexes of *wasted-ventilation* was then tested and included as a potentially independent variable into a multiple logistic regression model where the index of *wasted-ventilation* for the whole lung (global ROI) was already inserted. By backward selection of variables, we checked if regional information (indexes for one lung or quadrants) was adding independent information and could improve the performance of our prediction model. Model 1 is the multiple logistic regression model where only the index of *wasted-ventilation* for the whole lung (global ROI) is included, and Model 2 is the multiple logistic regression model where regional information (indexes for one lung or quadrants) was added. Receiver operating characteristic (ROC) curves for each tested model, along with their corresponding areas under the curve (AUC), were compared.

After electing the best prediction model according to the procedures above, our primary goal was to estimate its accuracy (with sensitivity and specificity estimated at the Youden-index–based optimal cutoff) in predicting perfusion defects experimentally produced by direct vascular occlusion (proximal or distal). Regional hypoperfusion that corresponds to areas of regional hypoventilation, such as that resulting from hypoxic pulmonary vasoconstriction, should not be classified as PE, regardless of its intensity.

As a preliminary validation of this prediction model for PE derived from piglets, we tested its sensitivity, specificity, and AUC performance in our database of 232 EIT-perfusion assessments obtained from 66 acute hypoxemic respiratory failure patients plus 56 EIT-perfusion assessments obtained from 10 CTEPH patients, with at least two assessments before and two after PTE per patient.

Finally, to evaluate the robustness of our elected model for PE, with more precise confidence intervals, and to account for the repeated measures design of our experimental study, we performed a bootstrap resampling (1000 resamples with replacement) of all our EIT-perfusion assessments (piglets and humans separately), with an inverse probability weighting based on the number of repeated measures per animal or per patient.

Pre-planned secondary outcomes were the correlation coefficient (Pearson), mean bias, and limits of agreement (LOA; Bland-Altman plots) between perfusion distribution estimated by EIT and CTPA. Although expecting a poorer correlation (related to dissimilar slice-thickness representation), we also aimed to test the agreement between perfusion distribution estimated by EIT and DCE-CT.

### Linear transformation

Before clinical validation, and to improve usability of the *wasted-ventilation* index, we used a linear transformation of the raw logistic regression equation obtained experimentally, bringing the optimal-Youden-cutoff as close as possible to zero, and simultaneously scaling it with minimum and maximum values in between − 10 and + 10. Such transformation applied to the index was a simple linear rescaling of the original weighted sum of regression coefficients. This approach does not alter the relative ordering of patients, nor does it affect the model’s discrimination or calibration properties, as it preserves all pairwise comparisons and proportional differences. The primary rationale for this transformation was to improve clinical usability by centering the score around 0 and constraining it to a more intuitive range (− 10 to + 10), facilitating bedside interpretation and threshold-based decision-making. Importantly, the transformation does not introduce bias, as it does not modify the relationship between predictors and outcome, but merely rescales the composite score. All performance metrics (e.g., AUC, calibration) remain unchanged under such linear transformations.

## Results

### Experimental data

Baseline characteristics of the ten animals of the new PE model are shown in Supplementary Material (Table S1). Six additional animals used to assess the effect of bronchial obstruction on V̇/Q̇ maps were reported elsewhere [16]. At baseline, EIT-estimated perfusion was evenly distributed between the right and left lungs. Proximal pulmonary artery occlusions markedly reduced perfusion to the *target ROI* (from 50.4 ± 4.2% to 9.2 ± 5.7%, *P* < 0.0001), whereas distal occlusions caused milder decreases (to 35 ± 9.0%, *P* < 0.0001).

Supplementary Material – Figure S1 illustrates EIT, DCE-CT, and CTPA perfusion analyses at baseline, during right proximal, and left distal occlusion. A strong correlation and good agreement were found between EIT and DCE-CT regional perfusion (Figures 2A–B). Agreement with CTPA *clot-burden* analysis was similar or superior (Figures 2C–D). Both modalities detected decreased perfusion in all 35 proximal occlusions. During distal occlusions, CTPA missed one of 32 cases, while EIT missed six, showing preserved perfusion (above 20% in all quadrants). Five out of 6 missing cases happened when the catheter was placed on the left ventral quadrant. In all missing cases, the *clot-burden* never exceeded 15%.

**Fig. 3:**
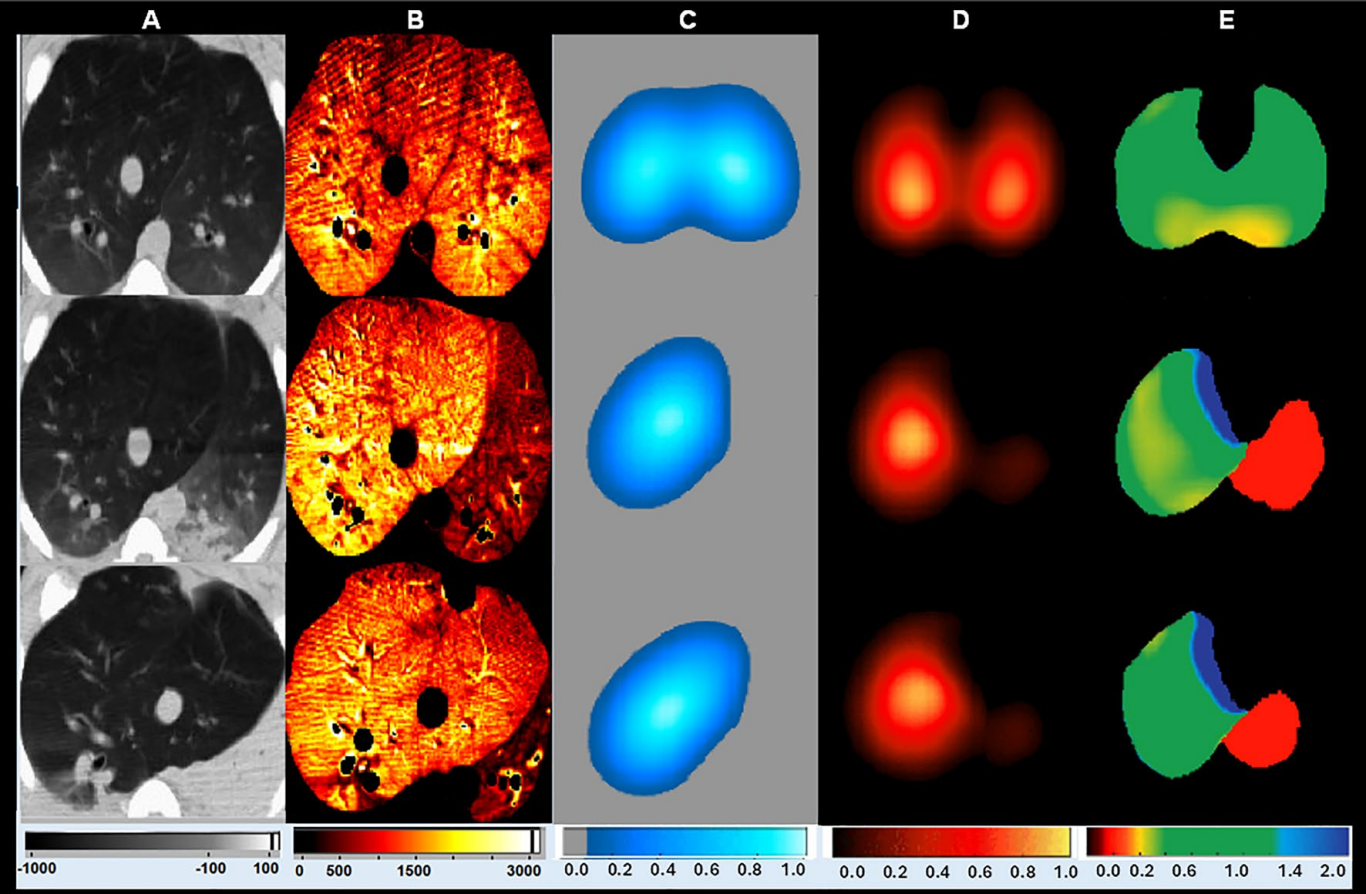
Representative images of bilateral ventilation and bronchial obstruction under fraction of inspired oxygen of 0.21 and 1.0. Computed tomography (CT) aeration images (Hounsfield units’ scale; (**A**); dynamic contrast-enhanced computed tomography perfusion images (normalized by density; (**B**); electrical impedance tomography (EIT) ventilation map (**C**); EIT perfusion map (**D**); EIT ventilation-perfusion (V̇/Q̇) map (E) are shown. Upper panel: with bilateral ventilation. Middle panel: with bronchial obstruction (left lung) and under fraction of inspired oxygen (F_I_O_2_) of 0.21. Bottom panel: with bronchial obstruction (left lung) and under F_I_O_2_ of 1.0. All images are from one representative piglet from an auxiliary study [16] aiming to assess regional pulmonary aeration, ventilation, perfusion, and V̇/Q̇ matching included in the current analysis. These additional piglets – not submitted to an experimental model of pulmonary embolism - also underwent a bronchial obstruction step, but under F_I_O_2_ of 0.21 and 1.0. They were studied during bilateral ventilation and with bronchial obstruction, with both F_I_O_2_, increasing our total number of *control cases* (i.e. without pulmonary artery occlusion). All these additional animals had a pulmonary artery catheter with a deflated balloon, therefore having negative gold-standard criteria for pulmonary embolism. Their experimental step with F_I_O_2_ of 1.0 (lower panel) during bronchial obstruction mimicked a condition of alveolar consolidation, like pneumonia. After bronchial obstruction of the left lung, EIT data demonstrates the absence of ventilation in the left lung and the shift of perfusion from left to right lung, likely caused by hypoxic vasoconstriction. During bronchial obstruction of the left lung, CT aeration (**A**) images show that the left lung is still aerated with 21% oxygen and totally collapsed with 100%. Notwithstanding that, both EIT V̇/Q̇ maps (**E**) (either 21% or 100%), show significant number of pixels belonging to the V̇/Q̇ < 0.5 compartment in the left lung (red pixels, i.e. pixels belonging to the *Mostly Perfused* EIT V̇/Q̇ compartment, EIT_LowV̇/Q_). This finding was consistent with pulmonary shunts of ~ 20% at both F_I_O_2_, demonstrating that the hypoxic vasoconstriction reflex was responsible for a decrease in regional perfusion to the left lung, but not strong enough to block all the perfusion that was still causing shunt within the left lung

Figure 3 shows DCE-CT and EIT V̇/Q̇ maps during bilateral ventilation and subsequent bronchial obstruction (F_I_O_2_ 0.21 and 1.0), revealing hypoxic vasoconstriction with hypoperfusion of the non-ventilated lung, used as control (non-occlusive) conditions.

### Physiological variables

The global *wasted-ventilation* index correlated positively with the ventilatory ratio (Supplementary Material – Figure S2) and alveolar dead space (Supplementary Material – Figure S3). During bronchial obstruction under F_I_O_2_ 1.0, EIT-measured perfusion in the non-ventilated ROI correlated positively with shunt fraction (Supplementary Material – Figure S4).

### Patient data

Seventy-six patients were studied, each undergoing three to five repeated measures, totaling 288 EIT perfusion assessments. Of these, 66 had acute hypoxemic respiratory failure (Supplementary Material – Table S2), with multiple EIT-perfusion studies performed along the time, eventually before and after thrombolysis. Ten additional patients had recently been diagnosed with CTEPH and were assessed both before and after PTE (Supplementary Material – Tables S3A-C).

## Regression models for predicting PE

### Experimental phase

**Fig. 4:**
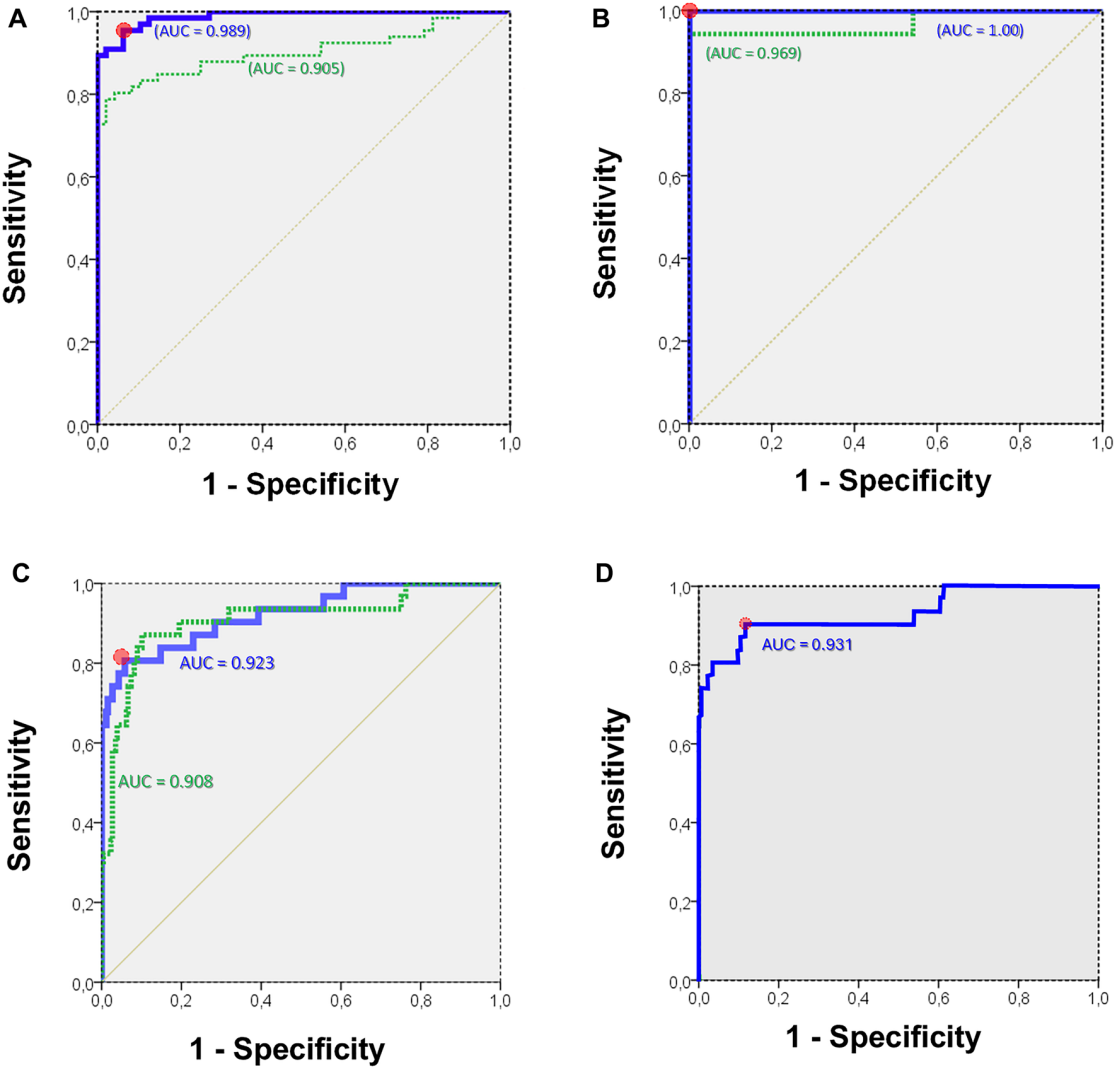
Receiver operating characteristic curves for assessing the accuracy of indexes of *wasted-ventilation* in discriminating cases with and without perfusion defects. Plots of the receiver operating characteristic (ROC) curve for: (**A**) Experimental data, having as the dependent variable pulmonary embolism (PE by reference method; proximal or distal). Model 1 (green, dashed line): global index of *wasted-ventilation*, i.e. ventilation directed to EIT_HighV̇/Q_ pixels, considering only the global region-of-interest (ROI) variable. Model 2 (blue, solid line): global plus local indexes of *wasted-ventilation*, i.e. ventilation directed to EIT_HighV̇/Q_ pixels for the global plus local ROIs variables. Optimal-Youden-cutoff (circle): sensitivity 96%, and specificity 94%. (**B**) Experimental data, having as the dependent variable only proximal PE (by reference method). Model 1 (green, dashed line): global index of *wasted-ventilation*, i.e. ventilation directed to EIT_HighV̇/Q_ pixels only for the global ROI variable. Model 2 (blue, solid line): global plus local indexes of *wasted-ventilation*, i.e. ventilation directed to EIT_HighV̇/Q_ pixels for the global plus local ROIs variables. Best cutoff (circle): sensitivity 100%, and specificity 100%. (**C**) Clinical data, having as the dependent variable PE (by reference methods). Model 1 (green, dashed line): global index of *wasted-ventilation*, i.e. ventilation directed to EIT_HighV̇/Q_ pixels only for the global ROI variable. Model 2 (blue, solid line): global plus local indexes of *wasted-ventilation*, i.e. ventilation directed to EIT_HighV̇/Q_ pixels for the global plus local ROIs variables. Optimal-Youden-cutoff (circle): sensitivity 81%, and specificity 94%. Values for each area under the ROC curve (AUC) are displayed in each corresponding line. (D) Clinical data, having as the dependent variable PE (by reference methods). Model 2 (blue, solid line): global plus local indexes of *wasted-ventilation*, i.e. ventilation directed to EIT_HighV̇/Q_ pixels for the global plus local ROIs variables. Now, with the Model calibrated in humans (combining forward and backwards selection of variables) and applied to humans. We found two almost equivalent best Optimal-Youden-cutoffs: sensitivity of 81% and specificity of 96%; or sensitivity of 91% with specificity of 88% (circle). Value for the area under the ROC curve (AUC) is displayed. EIT_HighV̇/Q_ = *Mostly Ventilated* electrical impedance tomography (EIT) ventilation-perfusion (V̇/Q̇) compartment within a ROI, in relation to the total ventilation received by that respective ROI

**Fig. 5:**
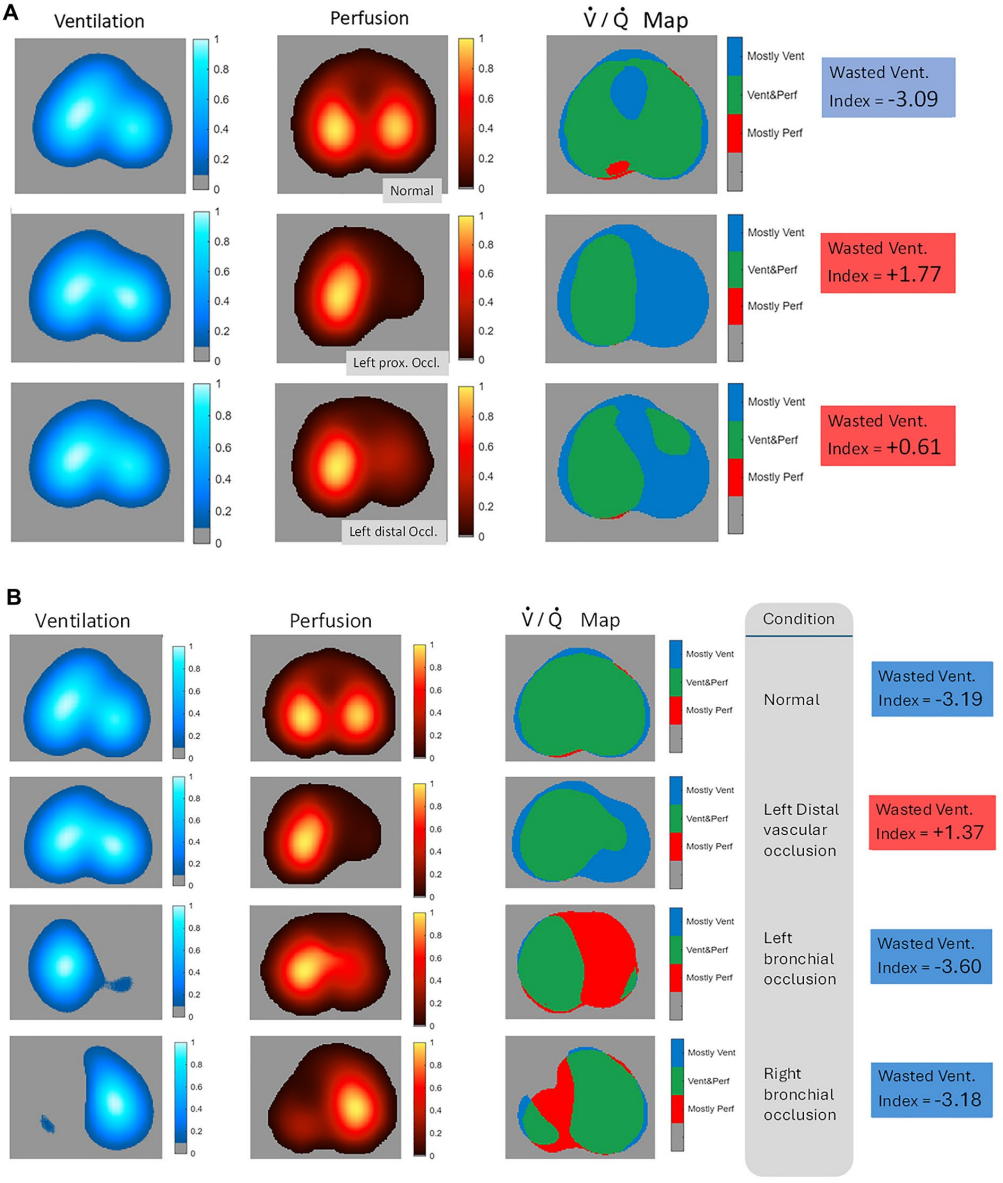
Ventilation, perfusion, and ventilation-perfusion maps by EIT from two representative piglets. (**A**) Electrical impedance tomography (EIT) ventilation, perfusion and ventilation-perfusion (V̇/Q̇) maps from one representative piglet from the experimental model of pulmonary embolism. The upper row shows the maps from the baseline condition, without any occlusion, whereas the middle and lower rows show the maps from the steps with proximal and distal pulmonary artery occlusion in the left lung. The corresponding indexes of *wasted*-*ventilation*, i.e. the percentage of ventilation directed to pixels classified as *Mostly Ventilated* within a region in relation to the total ventilation received by that respective region, are also shown. (**B**) EIT ventilation, perfusion and V̇/Q̇ maps from one representative piglet with the following conditions: normal (baseline, no occlusions), distal vascular occlusion in the left lung, bronchial occlusion of the left lung, and bronchial occlusion of the right lung. The corresponding indexes of *wasted*-*ventilation* are also shown. These maps contrast the situation of *true* vascular occlusion with the common situation of hypoxic vasoconstriction, typically associated with lung condensations found in pneumonia or atelectasis. Note that hypoxic vasoconstriction preserved negative *wasted-ventilation* indexes, despite the significant reductions in perfusion within the affected ROIs (mid panels, showing perfusion maps). Blue pixels in V̇/Q̇ map: ventilation directed to pixels classified as part of the *Mostly Ventilated* EIT V̇/Q̇ compartment (EIT_HighV̇/Q_), i.e. pixels with V̇/Q̇ > 2.0. Green pixels in V̇/Q̇ map: ventilation directed to pixels classified as part of the *Ventilated and Perfused* EIT V̇/Q̇ compartment (EIT_BalancedV̇/Q_), i.e. pixels with V̇/Q̇ between 0.5 – 2.0. Red pixels in V̇/Q̇ map: ventilation directed to pixels classified as part of the *Mostly Perfused* EIT V̇/Q̇ compartment (EIT_LowV̇/Q_), i.e. pixels with V̇/Q̇ < 0.5

Univariable logistic regression showed that the global *wasted-ventilation* index best predicted *clot-burden* (*P* < 0.0001; Fig. 4A). Multivariable regression identified five independent ROI variables (Global, Right, Left, LR, LL; Table S4), yielding superior discrimination of PE when included together in the regression model (*P* < 0.0001; 96% sensitivity, 94% specificity; Fig. 4A). The LR-ROI carried the second-largest coefficient, indicating that EIT_HighV̇/Q_ concentration in the LR quadrant increased specificity. Bootstrap resampling confirmed model stability (Supplementary Material – Table S5). In 35 proximal occlusions confirmed by CTPA, Model 1 reached AUC = 0.97 and Model 2 AUC = 1.0 (100% sensitivity and specificity; Fig. 4B). Figure 5 A illustrates sequential EIT changes from baseline to proximal and distal occlusion in piglets; Fig. 5B contrasts true vascular occlusion with hypoxic vasoconstriction, which preserved negative *wasted-ventilation* indexes. Ventilatory ratio performed worse (AUC = 0.68; Supplementary Material – Figure S5).

### Clinical phase

After the linear transformation described in the Methods, Models 1 and 2 were tested in patients. In the mixed cohort of 76 patients (Fig. 4C), Model 1 achieved AUC = 0.908 (*P* < 0.001; sensitivity 87%; specificity 90%), and Model 2 AUC = 0.923 (*P* < 0.001; sensitivity 81%; specificity 94%, optimal-Youden-cutoff = 0.0). Model 2 added independent predictive value (*P* = 0.001), correcting four false negatives and ten false positives found in Model 1. At 98% specificity for Model 2 (optimal-Youden-cutoff = + 2.2), sensitivity was 71% (positive likelihood-ratio, LR⁺ = 36); at 98% sensitivity for Model 2 (optimal-Youden-cutoff = − 2.4), specificity was 46% (negative likelihood-ratio, LR⁻ = 0.04).

One may hypothesize that our Model was not necessarily best calibrated for humans. To address that, we also performed a new regression analysis selecting only the human cases (see Figure S10 and Table S6 in the Supplementary Material).

Altogether, 31 EIT perfusion assessments were performed in patients with vascular obstruction and 257 in non-obstructed states. In seven out of eight patients with confirmed acute PE, the *wasted-ventilation* index was positive. In all three patients studied after thrombolysis, the *wasted-ventilation* index was completely reversed, with *all* values becoming negative after reperfusion (Supplementary Material – Figure S6). Ten CTEPH patients were assessed before and after surgery (Supplementary Material – Figure S6); nine had at least two paired studies. Post-thrombectomy indices decreased in all cases, although not enough to become fully negative in three out of 27 post-operative assessments.

Among CTEPH patients only, the *wasted-ventilation* index declined from + 5.94 [+ 1.63 to + 6.81] pre-PTE to − 2.76 [− 3.21 to − 2.24] post-PTE, with AUC = 0.96 and 87% correct classification - achieved with cutoff at zero. Bootstrap correction confirmed consistent accuracy and model coefficients (Supplementary Material – Table S5). Supplementary Material – Figure S7 shows typical EIT V̇/Q̇ maps from a control and an acute PE patient, while Supplementary Material – Figures S8 and S9A-B depict three typical CTEPH cases with marked postoperative index reductions.

## Discussion

This study assessed the accuracy of EIT V̇/Q̇ maps, together with a novel derived index of *wasted-ventilation*, for detecting pulmonary perfusion defects produced by vascular occlusion. To develop this index, we used a well-controlled experimental model of PE of varying intensities, in which EIT perfusion maps were compared against the procedurally imposed gold-standard vascular obstruction. We also compared EIT perfusion maps to those produced by DCE-CT. Furthermore, this study is the first to evaluate the agreement between EIT perfusion maps and the quantitative analysis of the *clot-burden* score derived from whole-lung CTPA images. The specificity of our *wasted-ventilation* index was further examined against varied clinical conditions of impaired lung perfusion in piglets, by creating perfusion defects not related to vascular occlusion, but caused by matched ventilation defects. Finally, after developing this *wasted-ventilation* index in piglets, we tested its performance in a mixed population of patients with acute respiratory failure or chronic thromboembolic pulmonary hypertension, of whom many had confirmation of PE by independent, gold-standard methods. The observed specificity and sensitivity suggest potential clinical applicability, for instance, in life-threatening situations where rapid “rule out” or “rule in” decisions for PE must be made especially under high-risk conditions of transportation.

In critical care patients, no diagnostic algorithm for PE has been fully validated, and the available diagnostic algorithms, such as the Wells and revised Geneva scores, remain unsatisfactorily inaccurate [20]. Intensive care patients under mechanical ventilation present frequent episodes of sudden hypoxemia from varied causes during their long stay, making it impossible to rule out PE on every single occasion of deterioration. Consequently, there is a potential overuse of CTPA, which unnecessarily exposes these patients to the inherent risks of ionizing radiation, iodinated contrast media, and risky intra- or inter-hospital transport. Additionally, many of these patients present with severe hypoxemia, shock, end-organ hypoperfusion, and eventually cardiac arrest, which often preclude the prompt realization of “standard” CTPA or invasive angiography [21], creating a challenging scenario where alternative diagnostic approaches are needed [3, 22, 23]. This is the situation where our new V̇/Q̇ assessment could be instrumental, especially for patients in whom immediate thrombolysis is being considered. According to our findings of sensitivity and specificity, a positive *wasted-ventilation* index could potentially increase by 13–36 times the post-test odds (bringing it close to 95%) of a true PE. This high likelihood ratio would be enough to improve emergency decisions at the bedside, with a performance equal or superior to point-of-care ultrasonography (POCUS) [24]. Conversely, very negative index values ( < − 2.4), observed in approximately 45% of our human cases, were associated with an approximately 25-fold reduction in post-test odds for PE-directed treatment (bringing it to < 5%), supporting the exclusion of high-risk thrombolysis and prompting consideration of alternative diagnoses.

Mechanically ventilated patients have a markedly higher risk for PE than non-intubated individuals [25, 26], creating a situation where alternative diagnostic tools for PE are very welcome. Recently, Girardi et al. found a 42% incidence of PE among critically ill patients undergoing CTPA for suspected embolism [27], compared with 20–25% positive scans in non-intubated patients with similar pre-test suspicion [28]. They also reported that transthoracic echocardiography – with signals of right-ventricular dysfunction - was the most suggestive of PE. Nevertheless, the diagnostic reliability of POCUS could only be acceptable when combined with other clinical findings, such as (i) no alternative diagnosis on chest radiography and (ii) D-dimer > 1000 ng/mL. With specificities ranging from 80 to 97% and sensitivities ranging from 22 to 80%, depending on the study and the POCUS signal to be considered, one of the major limitations of POCUS is the operator-dependent expertise required, and more so when an emergency evaluation is needed. This limitation frequently results in an insufficient negative likelihood ratio (> 0.2) to exclude PE, which necessitates confirmatory exams, such as a negative D-dimer or a negative lower-extremity venous Doppler, to rule out PE. When contrasted with the limited diagnostic accuracy of POCUS, the performance of the EIT-derived V̇/Q̇ scans presented here appears highly promising. An additional advantage of the proposed EIT index is its quantitative nature, as illustrated in Fig. 4, which suggests potential utility for monitoring therapeutic responses over time.

Recent case reports have suggested that asymmetric EIT-Q̇ or EIT-V̇/Q̇ maps could be used in suspected PE, when CTPA was not immediately available [11, 29, 30]. Our study extends those observations, by providing consistent and quantitative analyses in thrombolysed PE cases where the *wasted-ventilation* index correctly classified patients before (PE-positive) and after (PE-negative) therapy. Furthermore, the diagnostic performance remained high (AUC = 0.96) in ten patients with CTEPH, where the few false positives after thrombectomy seemed to reflect residual vascular disease, consistent with persistently elevated pulmonary pressures after surgery.

He et al. recently reported the first prospective EIT-based PE study using V̇/Q̇ maps, showing reasonable accuracy [12]. However, many limitations in their cohort were overcome by the present analysis: we presented a broader disease spectrum, we challenged patients with repeated V̇/Q̇ scans, under different PEEP levels and volemic status per patient, and we also included alternative diagnoses plus pre and post intervention conditions to enhance the specificity analysis. Interestingly, their proposed “dead-space” metric resembled our global *wasted-ventilation* index (Model 1) but lacks the regional information that strengthened our Model 2. Construction of Model 2 was only possible here through extensive experimental testing of proximal and distal occlusions together with alternative perfusion-impairment models.

Several limitations merit consideration. We assessed relative rather than absolute V̇/Q̇, assuming near-unity global V̇ and Q̇ with no regional dead-space variation [9, 14, 31, 32]. The presence of invasive monitoring for cardiac output is needed to obtain absolute values of pixel-level V̇/Q̇ mismatch [9, 31]. However, to date, fully validated methods to assess only EIT-based absolute physiological V̇/Q̇ ratio noninvasively and at the bedside and are still lacking. Promisingly, data from some preliminary proof-of-concept studies have proposed possible approaches to obtain surrogate measures for cardiac output in EIT V̇/Q̇ matching assessments [14, 32]. We think that such possibility may further strengthen the physiologic interpretation and future development of our proposed V̇/Q̇ index. A low pixel cutoff (5%) improved detection of subtle shunt regions at the expense of signal-to-noise ratio but proved adequate. The quasi-3D nature of EIT may reduce sensitivity for distal thrombi while favoring detection of proximal emboli. Our contrast-EIT perfusion assessments involved breath-holding during the saline injection and that may alter perfusion dynamics. On this regard, Victor et al. assessed non-apnea contrast-EIT in animal models and two acute respiratory distress syndrome patients, showing strong agreement between the two methods [33]. And very recently, Gao et al. proposed a simple method with low-pass filtering to avoid the breath-holding. They conducted a randomized trial to investigate the consistency of contrast-EIT with and without breath-holding in a cohort of mechanically ventilated patients in the ICU [34]. They showed that the non-apnea contrast-EIT method is feasible in mechanically ventilated ICU patients, demonstrating good consistency with the conventional apnea method. However, among other limitations of the study, they did not investigate patients with specific pulmonary pathologies, where the filtering performance may differ. In addition, considering the physiological differences between apnea and non-apnea methods, further validation against gold standards such as SPECT is required. We think that a fully validated method avoiding the breath-holding may potentially offer an even more physiologically realistic assessment of lung perfusion and expand the clinical applicability of our proposed V̇/Q̇ index, further strengthening the physiological interpretations and future developments of our proposed index. Given the limited prior literature to inform sample size estimation, we included a convenience sample of all patients at our center who underwent perfusion assessments with EIT and had a definitive clinical diagnosis of either positive or negative pulmonary embolism. In this sample, the number of positive cases was relatively small. Despite the reasonable precision of the diagnostic estimates, this may limit the immediate generalizability of our findings and highlights the need for further validation in future studies. Also, the reference standard for PE-negative classification was not fully homogeneous across patients. Consequently, we cannot exclude the possibility that some patients classified as PE-negative had uncertain or small pulmonary emboli that were not definitively confirmed. But our definition of PE-negative cases in our clinical cohort reflects the realities of clinical practice, particularly in critically ill or unstable patients, where it is often not feasible to apply the same confirmatory diagnostic test to all individuals. Finally, we think that future studies should also compare EIT directly with other bedside modalities, such as echocardiography.

## Conclusions

In conclusion, this translational study supports EIT as a promising bedside technique for detecting pulmonary arterial occlusions of varying severity and distinguishing them from other causes of perfusion impairment. The proposed *wasted-ventilation* index for PE detection demonstrated accuracy compatible with clinical use, providing foundation for further investigation. The strong agreement between EIT and established imaging modalities (CTPA, DCE-CT) indicates its potential as an alternative when CTPA is impractical, particularly in high-risk patients. Prospective studies are warranted to validate these results and define EIT’s clinical utility relative to other bedside methods.

## Electronic Supplementary Material

Supplementary Material 1

## Data Availability

The datasets used and/or analyzed during the current study are available from the corresponding author on reasonable request.

## Abbreviations

V̇/Q̇: Ventilation-perfusion
PE: Pulmonary embolism
CTPA: Computed tomography pulmonary angiography
EIT: Electrical impedance tomography
DCE-CT: Dynamic contrast-enhanced computed tomography
F_I_O_2_: Fraction of inspired oxygen
ΔZ: Tidal impedance changes
CPAP: Continuous positive airway pressure
PEEP: Positive end-expiratory pressure
ROI: Region-of-interest
UR: upper-right
UL: upper-left
LR: lower-right
LL: lower-left
EIT_HighV̇/Q_: *Mostly Ventilated* EIT V̇/Q̇ compartment, i.e. comprising pixels with V̇/Q̇ > 2.0
EIT_BalancedV̇/Q_: *Ventilated and Perfused* EIT V̇/Q̇ compartment, i.e. comprising pixels with V̇/Q̇ between 0.5 – 2.0
EIT_Low_V̇/Q: *Mostly Perfused* EIT V̇/Q̇ compartment, i.e. comprising pixels with V̇/Q̇ < 0.5
CTEPH: Chronic thromboembolic pulmonary hypertension
SPECT: Single-photon emission computed tomography
PTE: Pulmonary thromboendarterectomy
ROC: Receiver operating characteristic
AUC: Area under the receiver operating characteristic curve
LOA: Limits of agreement
LR⁺: Positive likelihood-ratio
LR⁻: Negative likelihood-ratio
POCUS: Point-of-care ultrasonography
