# Supplementary material for: Accuracy of electrical impedance tomography to detect perfusion defects in pulmonary embolism
Source: Crit Care. 2026 May 20;30:283. doi: 10.1186/s13054-026-06073-y (PMC13224680; doi:10.1186/s13054-026-06073-y)
Supplement: Supplementary file 1 — Supplementary Material 1 [file 13054_2026_6073_MOESM1_ESM.docx]

**Accuracy of Electrical Impedance Tomography to Detect**

**Perfusion Defects in Pulmonary Embolism**

Eduardo Augusto Pinto Rodrigues, Eder Chaves Pacheco, Maria Aparecida Miyuki Nakamura, Ana Carolina Cardoso dos Santos, Jade Lara de Melo, Glauco M. Plens, Glasiele Cristina Alcala, Marcus Victor, Yi Xin, Maurizio Cereda, Susimeire Gomes, Hye Ju Lee, Bruno M. Ribeiro, Rafael Moraes Ianotti, Orival Freitas-Filho, Jose Leonidas Alves-Jr, Lorenzo Berra, Caio C. A. Morais, Larissa Bertacchini, Mikuláš Mlček, Rogerio Souza, João Batista Borges **⃰**, Eduardo L. V. Costa, Marcelo B. P. Amato **⃰**

**⃰** Corresponding authors:

João Batista Borges, M.D., Ph.D., Institute of Physiology, 1^st^ Faculty of Medicine, Charles University, Prague, Czech Republic. Albertov 5, Prague, 128 00.

E-mail: [joaobatistaborges8@gmail.com](mailto:joaobatistaborges8@gmail.com)

Marcelo B. P. Amato, M.D., Ph.D., Laboratorio de Pneumologia LIM 09 Faculdade de Medicina da Universidade de Sao Paulo. Av. Doutor Arnaldo, 455 (Sala 2144, 2nd Floor), Sao Paulo 01246-903, Brazil.

E-mail: [marcelo.amato@fm.usp.br](mailto:marcelo.amato@fm.usp.br)

**Supplementary Material**

**Methods**

**Experimental Data**

The Ethics Committee for Experimental Studies approved the study (No. 1434/2020, Faculdade de Medicina da Universidade de São Paulo). All procedures were performed in accordance with national laws regarding the use of animals for research. Ten healthy female Landrace piglets (34.9 ± 2.6 kg) were intubated, anesthetized with a continuous infusion of ketamine (5 mg/kg/h), fentanyl (1.5 mcg/kg/h), and pancuronium (0.1 mg/kg/h), and mechanically ventilated with a Nihon Koden ventilator NKV 550 (Nihon Kohden, Tokyo, Japan), from which end-tidal carbon dioxide (ETCO_2_) and volumetric capnography parameters were monitored and acquired. One femoral artery was cannulated to monitor arterial blood pressure and collect arterial blood gas samples. A pulmonary artery catheter was introduced in the right jugular vein to measure pulmonary artery pressure and cardiac output (thermodilution method). The left jugular vein was cannulated for medications and electrical impedance tomography (EIT) perfusion measurements. Peripheral capillary oxygen saturation (SpO_2_), heart rate, electrocardiogram, and arterial blood pressure were monitored using a multiparameter monitor Nihon Kohden Life Scope Triton BSM-6701 (Nihon Kohden, Tokyo, Japan). From the preparation of the animal, the EIT device continuously monitored and acquired impedance, airflow, and airway pressure measurements at 50 Hz through a 32-electrodes belt and a proximal dedicated pressure and flow sensors.

After the initial ventilation, a lung recruitment maneuver (LRM) was performed, and an arterial blood sample was drawn to exclude animals with PaO_2_ + PaCO_2_ ≤ 400 mmHg. After the piglets were prepared in the intensive care unit (ICU) of the Laboratory of Medical Investigations of Pulmonology, they were transferred to the computed tomography (CT) scan acquisition room. Animals were then stabilized for 15 minutes prior to image acquisitions.

They stayed in the supine position throughout the study. The animals were euthanized at the end of the study with boluses of sedatives followed by a 10 mL 19.1% KCl bolus.

Experimental Outline

Perfusion assessments were acquired using EIT, dynamic-contrast enhanced computed tomography (DCE-CT), and computed tomography pulmonary angiography (CTPA) at the following steps and order: baseline (without any occlusion), after inducing a proximal pulmonary artery occlusion by inflating the balloon of the pulmonary artery catheter at a proximal level, and after inducing a distal pulmonary artery occlusion by inflating the balloon of the pulmonary artery catheter at a distal level. This sequence of steps was performed for both right and left lungs, starting with the lung into which the pulmonary artery catheter was placed during the preparation of the animal in the intensive care unit of the laboratory. In the event of hemodynamic instability, the pulmonary artery occlusion was promptly halted.

The sequence of imaging acquisitions, made for each step described above, was the following: EIT, DCE-CT, EIT, and CTPA. The repeated EIT acquisitions were performed to ensure that the step condition was stable throughout the CT acquisitions. The EIT data used for the final analysis were taken from the average of the two.

To be considered satisfactory, the following criterion was used to ensure that a situation of either proximal or distal pulmonary artery occlusion was temporarily achieved: a clear transition of tracings from pulmonary artery waveforms to wedged waveforms. This criterion was also considered as our gold-standard for vascular occlusion for our receiver operating characteristic (ROC) curve and accuracy analysis of the animals. Each situation of vascular occlusion was kept for 15 minutes at the end of which a new arterial blood gas was collected.

After the pulmonary artery occlusions phase, a new LRM was performed, and a bronchial obstruction step was added to these piglets. Such bronchial obstruction was induced by selectively intubating the right or left lung and surgically sealing the trachea around the endotracheal tube, and it was promptly confirmed by the real-time ventilation distribution of EIT. Previously to the bronchial obstruction, the animal was ventilated with fraction of inspired oxygen (F_I_O_2_) of 0.21 for 15 minutes. This procedure ensured that a high nitrogen content was present in the gas trapped behind closed airways, thus creating local hypoventilation and hypoxia, but avoiding massive reabsorption atelectasis. This local hypoventilation and hypoxia was expected to produce hypoxic pulmonary vasoconstriction, thus creating a scenario of matched impairment of ventilation and perfusion within one entire lung. After 15 minutes of bronchial obstruction, new perfusion assessments by EIT and DCE-CT were acquired. A pair of arterial and mixed venous blood gas samples were also collected.

To enrich our dataset, the present analysis further included data from another study recently performed in our laboratory [1]. These additional piglets (six) were not submitted to an experimental model of pulmonary embolism (PE), but underwent bronchial obstruction steps, creating 3 study conditions per animal: a) bilateral ventilation; b) bronchial obstruction performed under F_I_O_2_ of 0.21; and c) bronchial obstruction performed under F_I_O_2_ of 1.0. This inclusion increased the number of “challenging” control cases (cases with matched hypoperfusion and hypoventilation, but without pulmonary artery occlusion). All these additional animals had a pulmonary artery catheter with a deflated balloon and had negative gold-standard criteria. The use of F_I_O_2_ of 1.0 during bronchial obstruction mimicked a condition of alveolar consolidation.

Mechanical Ventilation

After a stabilization period of 15 minutes in the CT room, the positive end-expiratory pressure (PEEP) of each animal was individually optimized before lung imaging acquisitions: after a LRM, an EIT-based decremental PEEP titration [2] was performed under volume-controlled mode, tidal volume of 5 mL/kg, respiratory rate of 25 bpm, F_I_O_2_ of 1.0, and inspiratory/expiratory ratio of 1:2. The PEEP levels were decreased in fixed intervals of 2 cmH_2_O, from 24 to 4 cmH_2_O, 30 seconds in each level. The EIT-based optimum PEEP (PEEP_EIT_) was defined as the lowest PEEP level in which lung collapse was < 1%.

After a new LRM, the animals were ventilated throughout the protocol in volume-controlled mode, tidal volume 8 mL/kg, respiratory rate of 25 bpm, F_I_O_2_ to maintain SpO_2_ > 93%, PEEP_EIT_, and inspiratory/expiratory ratio of 1:2.

For shunt calculations during bronchial obstruction and one-lung hypoxia, an F_I_O_2_ of 1.0 was applied in the contralateral lung for 15 minutes before the corresponding blood sampling (arterial and mixed venous).

Imaging Studies

*EIT*

EIT continuously measured thoracic impedance variations at a rate of 50 Hz (Enlight 2100, Timpel Medical, Sao Paulo, Brazil). Extra-small electrode bands (60 mm-thick) were placed inferior to the piglet’s glenohumeral joint according to the manufacturer’s recommendation. A pneumotachograph was placed proximally to the endotracheal tube and connected to the EIT monitor. The data were then reconstructed into an image consisting of a 32 by 32-pixel spatial matrix [3]. Ventilation data were obtained by measuring tidal impedance changes (ΔZ) for each pixel, averaged over 10 breaths. ΔZ correlates well with CT measures of tidal volume [4]. EIT ΔZ will be referred to as EIT ventilation.



EIT lung perfusion was conducted in each tested condition as previously described [5]. A bolus of 10% sodium chloride solution (10 mL) was injected over one second into a central venous catheter during 20 seconds of apnea in continuous positive airway pressure (CPAP) mode, using the PEEP level measured in the ventilator before the apnea. The impedance versus time waveform of each pixel was acquired and exported to a custom LabVIEW software (National Instruments, Austin, TX). Each pixel’s impedance versus time curve was analyzed for a biphasic model as previously described [5]. If two components were present, the first appearing component indicated pre-lung signal from the right heart phase or vascular tissue. The resulting lung curve was fitted to a single gamma function which was then reconstructed into a 32 by 32-pixel matrix overlapping the ventilation matrix. Using the first-pass kinetic method [5], the maximum slope of each pixel’s gamma function was calculated yielding a relative regional perfusion map.

*EIT Post-processing*

For each individual animal, EIT maps of ventilation and perfusion were normalized to obtain a maximum pixel value of 1.0 within each map. Pixels with values less than 5% of the maximum were excluded. Next, each animal’s ventilation and perfusion maps were expressed as one (global), two (right and left lung), and the following four quadrants’ regions-of-interests (ROIs): upper-right (UR), upper-left (UL), lower-right (LR), and lower-left (LL). Pixel-wise ventilation and perfusion values were summed within each ROI and divided by the sum of the global map, yielding percent values of regional ventilation and perfusion directed to each ROI.

By comparing pixel-wise ventilation and perfusion maps, we attributed to each pixel a ventilation-perfusion (V̇/Q̇) matching value, generating EIT V̇/Q̇ maps.

For each EIT V̇/Q̇ map, three V̇/Q̇ compartments were computed:

- *Mostly Ventilated* (EIT***_HighV̇/Q̇_***): Pixels with V̇/Q̇ > 2.0.
- *Ventilated and Perfused* (EIT***_BalancedV̇/Q̇_***): Pixels with V̇/Q̇ between 0.5 – 2.0.
- *Mostly Perfused* (EIT***_LowV̇/Q̇_***): Pixels with V̇/Q̇ < 0.5.

Finally, within each ROI (including the global ROI or global V̇/Q̇ map), we computed the regional index of *wasted-ventilation*: the percentage of ventilation directed to pixels classified as *Mostly Ventilated* within the ROI*,* in relation to the total ventilation received by that respective ROI.

*DCE-CT*

DCE-CT was obtained using a Siemens Somaton® Emotion 16 multislice computed tomography equipment (Siemens AG®, Germany). For the evaluation of DCE-CT, acquisition parameters were the following: voltage of 80 kV, 110 mA of current, 0.6 seconds rotation time, and a square matrix of 512 x 512 pixels. The acquisition was made in a region above the diaphragm with a total thickness of 19.2 mm, corresponding to 4 tomographic sections (blocks) of 4.8 mm, with a reconstruction increment of 0.05 seconds. The acquisition was made during an apnea of 30 seconds in CPAP mode (using the PEEP level measured in the ventilator before the apnea) with an injection of 20 mL of a nonionic iodinated contrast in the right atrium (2 seconds of injection time) during the acquisition of 20 seconds of a dynamic CT, beginning 3-5 seconds before the contrast injection.



The pulmonary blood flow images were obtained using the ‘steepest slope’ method [6, 7]. In short, the first-pass kinetic traces for all segmented lung tissue voxels and for the pulmonary artery signal (as the input function) were fitted to a gamma variant function. The pulmonary blood flow was then calculated as the maximum rising slope divided by the peak intensity of the pulmonary artery signal [5, 6]. The DCE-CT data were measured in mL/min and expressed as a percentage of total perfusion.

To compare the regional distribution of perfusion by DCE-CT versus EIT, at each step, each animal’s perfusion map (DCE-CT and EIT) was divided into two ROIs: right and left lung. The ROI where the tip of the Swan-Ganz catheter was located (or where we performed the bronchial obstruction) was called *target ROI*.

*CTPA*

CTPA was also obtained using the Siemens Somaton® Emotion 16 multislice computed tomography equipment (Siemens AG®, Germany) used for the DCE-CT acquisitions. Iodinated contrast (1 mL/kg) at a flow rate of 8 mL/s was injected, and images with slice thickness of 0.625 mm were obtained as soon as the contrast agent reached the main pulmonary artery.

CTPA data were quantified after image analysis by a cardiothoracic radiologist unaware of the EIT results. Perfusion distribution by CTPA, estimated for the *target ROI*, was calculated as follows (*clot-burden*):

$Perfusion distribution = \frac{remaining number of patent vessels within target ROI}{total number of patent vessels within whole lung}$ *(1)*

Figure 1 shows an illustrative case of this *clot-burden* quantification. Only segmental arteries previously patent at baseline were counted.

Similarly to the analysis performed for DCE-CT, the comparison between CTPA versus EIT considered only two ROIs, right and left lung, where one of them was the *target ROI* for vascular or bronchial obstruction.

Physiological Variables

The following physiological variables were also computed for each step: Ventilatory Ratio, ETCO_2_ and alveolar dead-space (from the volumetric capnography), respiratory mechanics, hemodynamic parameters, and gas exchange parameters (including shunt).

**Patient Data**

Patients monitored by EIT as part of routine care or as part of a research protocol [8] formed our validation sample with 232 EIT-perfusion studies obtained from sixty-six patients with acute hypoxemic respiratory failure plus 56 EIT-perfusion studies obtained from ten patients with chronic thromboembolic pulmonary hypertension (CTEPH) [9, 10]. Patients typically performed three to five repeated EIT-perfusion studies along the stay, totaling 288 EIT perfusion assessments. At least two EIT-perfusion assessments were performed before and two after pulmonary thromboendarterectomy (PTE) per patient.

CTEPH is caused by persistent organized thromboembolic obstruction of the pulmonary arteries from incompletely resolved acute PE [11, 12]. In all studies, a bolus of 7.5% or 10% sodium chloride solution (10 mL) was injected over < 2 seconds into a central venous catheter during apnea (20 seconds) under CPAP mode, using the PEEP level measured before the apnea. Many patients had various EIT-perfusion measurements repeated over an interval of less than 24 hours and under different clinical conditions: before and after fluid resuscitation, before and after PEEP increase, before and after thrombolysis (in those with confirmed PE), or before and after PTE, which is the definitive treatment and is potentially curative for CTEPH [9, 10, 13].

Patients were monitored with the Enlight 2100 EIT at the respiratory ICU or the post-operative surgical ICU, both at Incor/HC-FMUSP, University of Sao Paulo, Sao Paulo, Brazil. Additional patients were monitored at the MGH (Harvard University) in Boston as part of the Lung Rescue Team routine [14]. All patients presented diagnosis of acute hypoxemic respiratory failure or CTEPH at the time of the EIT V̇/Q̇ study.

The approvals by the ethical committees are the following:

- Acute hypoxemic respiratory failure patients: 1) CAAE 90728718.7.0000.0068 / Decision Number 3.690.968; 2) CAAE: 15026419.1.0000.0068 / SDC 4843/19/062; 3) Multicenter prospective physiological study NCT04460859 / approved by each center’s research ethics board; 4) Study approved by the Mass General Brigham Institutional Review Board (Protocol number #2020P003196).
- CTEPH patients: CAAE 78916224.4.0000.0068 / Decision Number 6.819.396.

EIT perfusion studies were pre-planned according to protocol design or indicated by the attending physician because of a high suspicion of PE (a combination of D-dimer > age-adjusted-cutoff and Geneva score > 10, found in 18 cases) and the impossibility of immediate transportation to CT or single-photon emission computed tomography (SPECT) machines. See above the respective approvals by the ethical committees.

The diagnosis of PE was confirmed in 7 of 18 patients with acute hypoxemic respiratory failure and high clinical suspicion, based on CTPA or pulmonary perfusion SPECT. An additional hypoxemic patient had PE identified incidentally on CT performed at ICU admission. For PE-negative cases, patients met one of two criteria: (a) absence of clinical suspicion with negative non-invasive rule-out tests [15, 16] indicating low pre-test probability, or (b) non-confirmatory CTPA or perfusion mapping, regardless of pre-test probability or non-invasive assessments. The latter applied to the remaining 11 patients with hypoxemic respiratory failure and high clinical suspicion.

The added patients with CTEPH were submitted to PTE at least six months after the latest related clinical event and at least three months of adequate anticoagulation.

**Multiple Logistic Regression Models and Statistical Analysis**

In pilot studies performed in our laboratory, we realized that the global index of *wasted-ventilation* (ventilation directed to EIT***_HighV̇/Q̇_*** pixels for the global ROI) correlated well with large *clot-burdens*. However, smaller *clot-burdens* (commonly produced by distal balloon occlusions), which often produced localized clusters of EIT***_HighV̇/Q̇_*** pixels, were commonly missed. To enhance sensitivity, we calculated additional indexes of *wasted-ventilation* within each of the smaller ROIs, testing whether their inclusion into multiple logistic regression models could enhance our performance to detect PE. Each of those regional indexes of *wasted-ventilation* was then tested and included as potentially independent variable into a multiple logistic regression model where the index of *wasted-ventilation* for the whole lung (global ROI) was already inserted. By backward selection of variables, we checked if regional information (indexes for one lung or quadrants) was adding independent information and could improve the performance of our prediction model. Namely, Model 1 is the multiple logistic regression model where only the index of *wasted-ventilation* for the whole lung (global ROI) is included; and Model 2 is the multiple logistic regression model where regional information (indexes for one lung or quadrants) were added. ROC curves for each tested model with corresponding areas under the curve (AUC) were compared.

After electing the best prediction model according to the procedures above, our primary goal was to estimate its accuracy (with sensitivity/specificity) in predicting perfusion defects experimentally produced by direct vascular occlusion (proximal or distal). Regional hypoperfusions, whatever its intensity, caused by hypoxic pulmonary vasoconstriction should not be classified as PE.

As a preliminary validation of this prediction model for PE derived from piglets, we tested its sensitivity, specificity, and AUC performance in our data bank of 232 EIT-perfusion studies obtained from 66 patients with acute hypoxemic respiratory failure plus 56 EIT-perfusion studies obtained from 10 patients with CTEPH.

Finally, to evaluate the robustness of our elected model for PE, with more precise confidence intervals, and to account for the repeated measures design of our experimental study, we performed a bootstrap resampling (1000 resamples with replacement) of our 114 EIT-perfusion studies, with an inverse probability weighting based on the number of repeated measures per animal. The same bootstrap resampling procedure was also performed for the patient data.

Pre-planned secondary outcomes were the correlation coefficient (Pearson), mean bias, and limits of agreement (Bland-Altman plots) between perfusion distribution estimated by EIT and CTPA. Although expecting a poorer correlation (related to dissimilar slice-thickness representation), we also aimed to test the agreement between perfusion distribution estimated by EIT and DCE-CT.

Data are expressed as mean ± standard deviation or median [IQR] when appropriate. Significance was defined as α ≤ 0.05. All analyses were conducted using R 4.2.2.

**Linear Transformation**

Before clinical validation, and to improve usability of the *wasted-ventilation* index, we used a linear transformation of the raw logistic regression equation obtained experimentally, bringing the optimal-Youden-cutoff as close as possible to zero, and simultaneously scaling it with minimum and maximum values in between -10 and +10. Such transformation applied to the index was a simple linear rescaling of the original weighted sum of regression coefficients. This approach does not alter the relative ordering of patients, nor does it affect the model’s discrimination or calibration properties, as it preserves all pairwise comparisons and proportional differences. The primary rationale for this transformation was to improve clinical usability by centering the score around 0 and constraining it to a more intuitive range (−10 to +10), facilitating bedside interpretation and threshold-based decision-making. Importantly, the transformation does not introduce bias, as it does not modify the relationship between predictors and outcome, but merely rescales the composite score. All performance metrics (e.g., AUC, calibration) remain unchanged under such linear transformations.

**Results**

**The Characteristics of the False-positive Cases**

We had 17 cases of false positive results when using the Youden cutoff (with specificity of 94%). However, when applying the threshold optimized for specificity, the number of false positive was just 4 cases. At the Youden cutoff, we had:

-5 cases (within 3 patients) related to post-operative cardiac surgery.

-3 cases (within 2 patients) with extreme obesity and acute hypoxemic respiratory failure.

-4 cases (within 2 patients) with severe COVID-19 intubated.

-5 cases of postoperative surgery for CTEPH (in which they might have residual disease).

When using the cutoff optimized for high specificity, the false positive cases were:

-2 cases (within one single patient) with post-operative cardiac surgery.

-2 cases (within one single patient) with sever COVID-19, intubated.

**Figures**


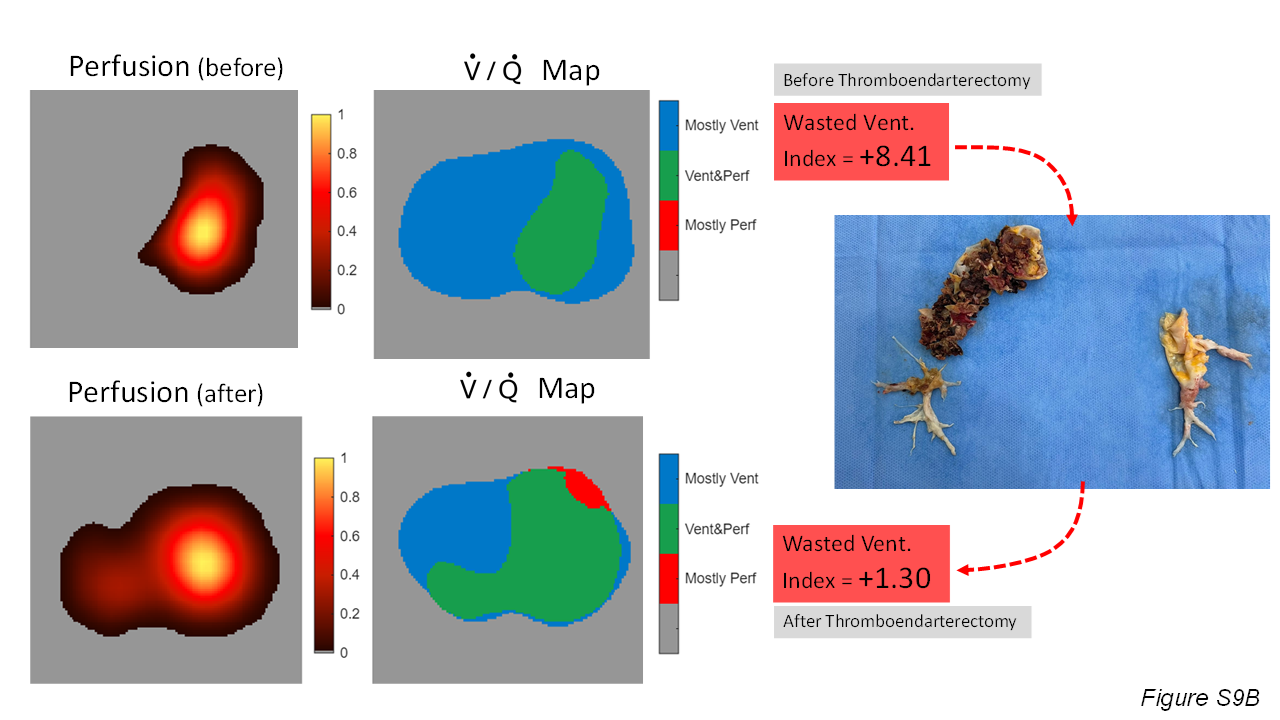

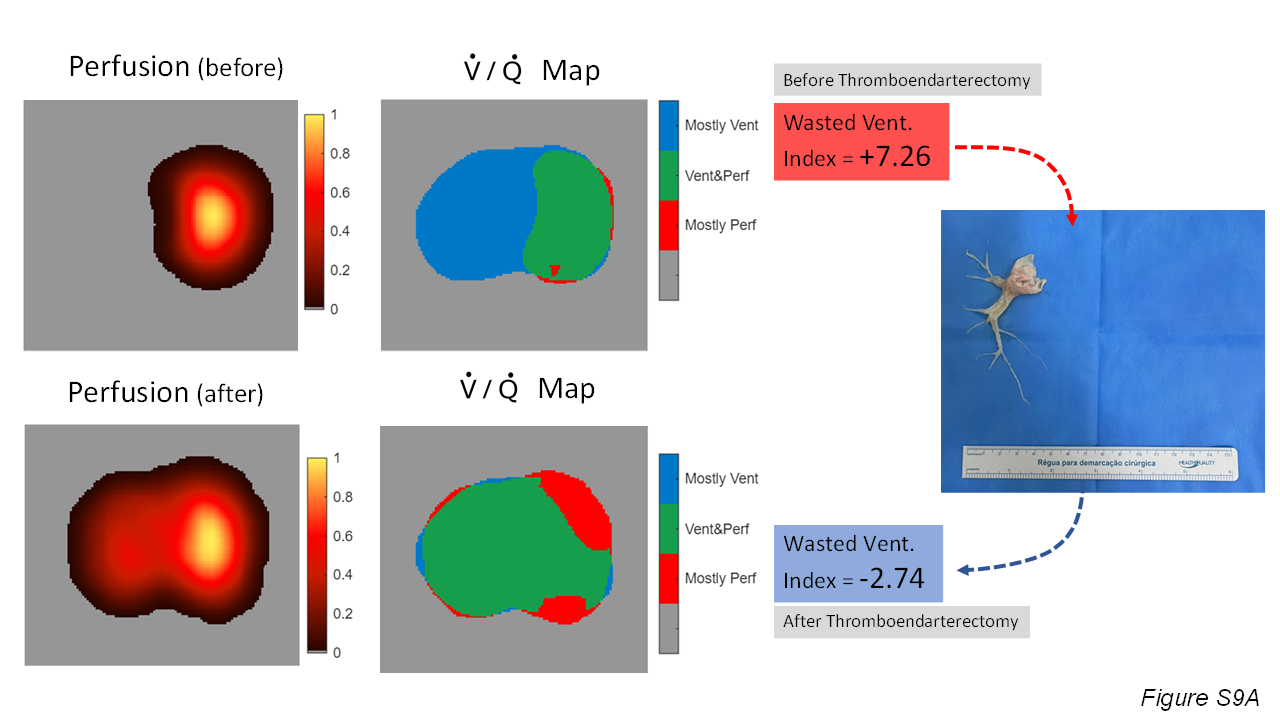

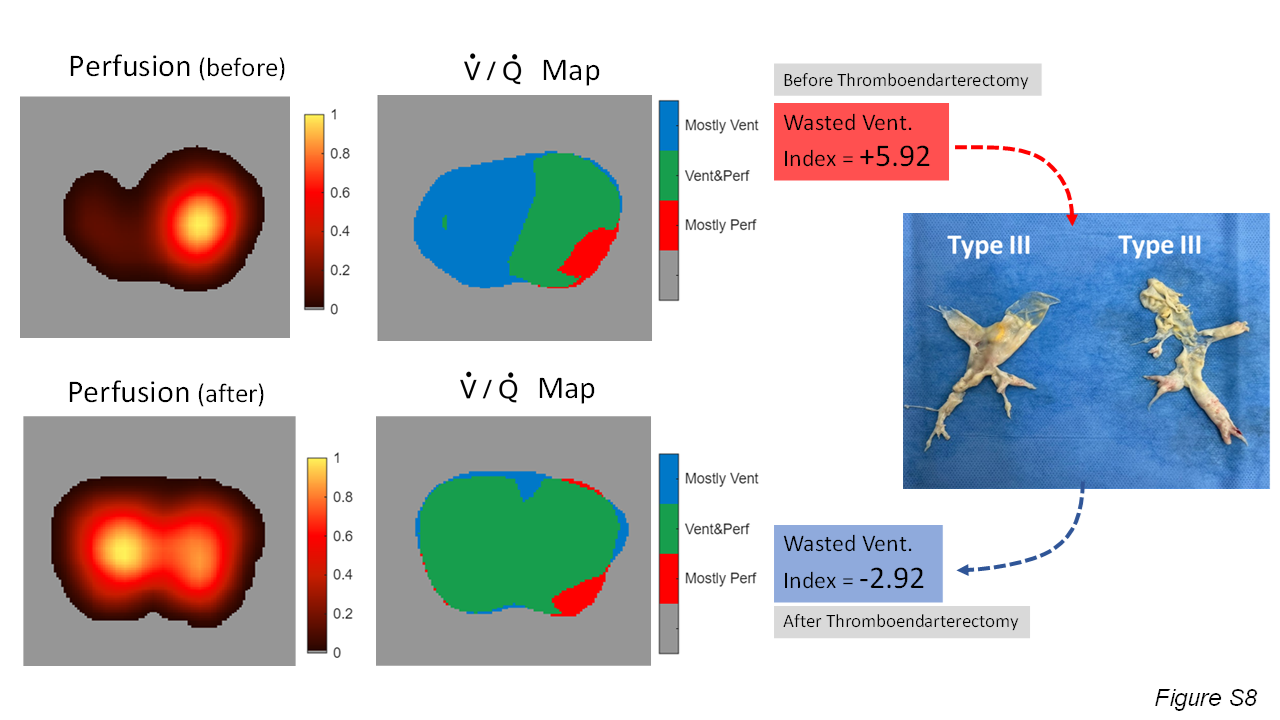

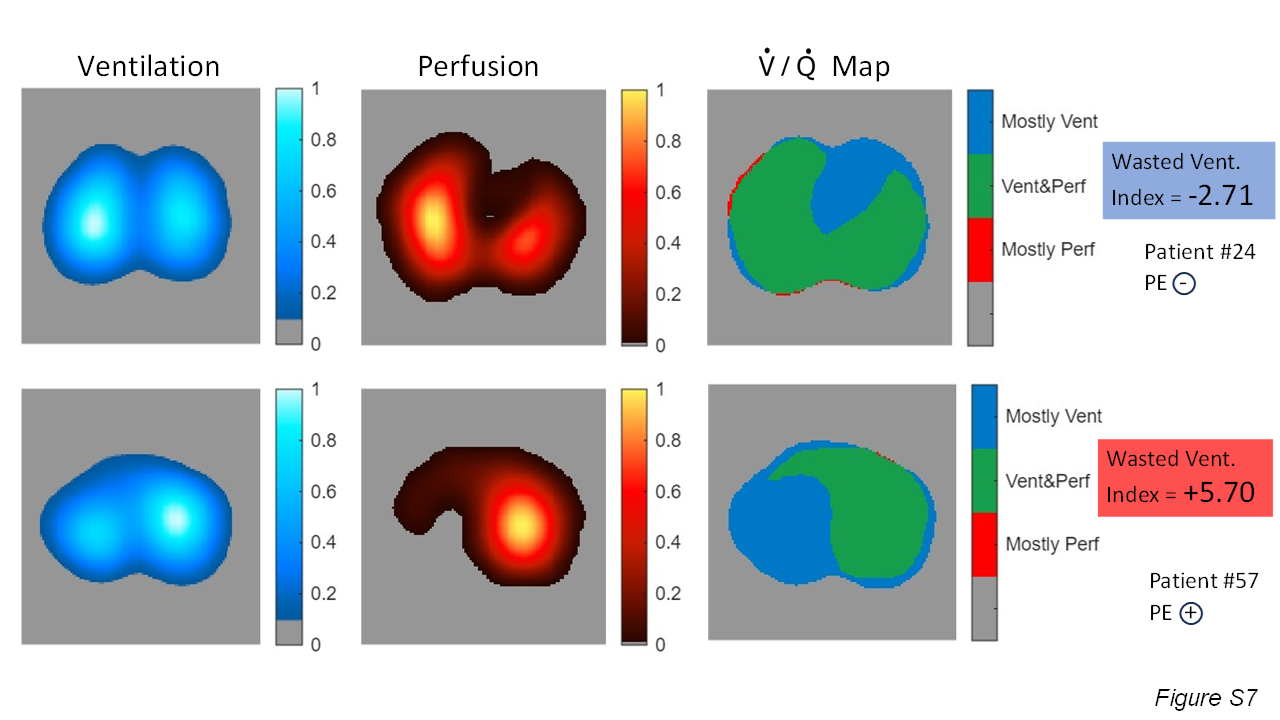

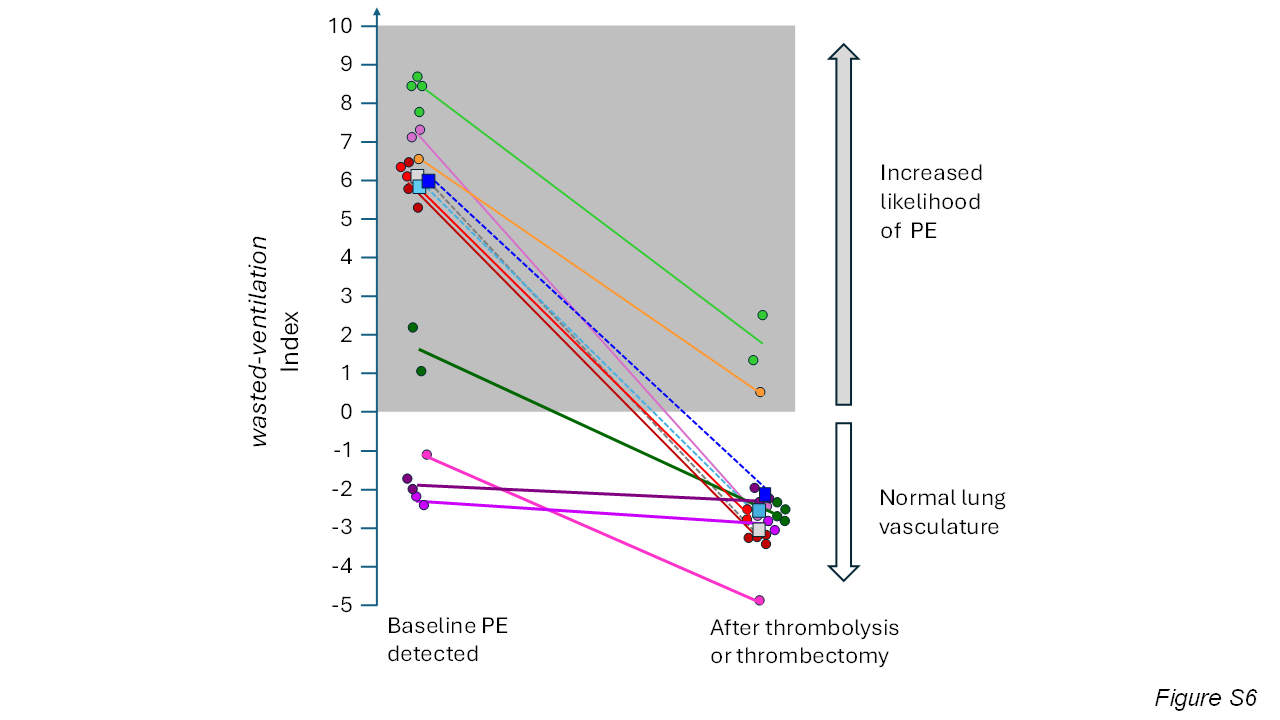

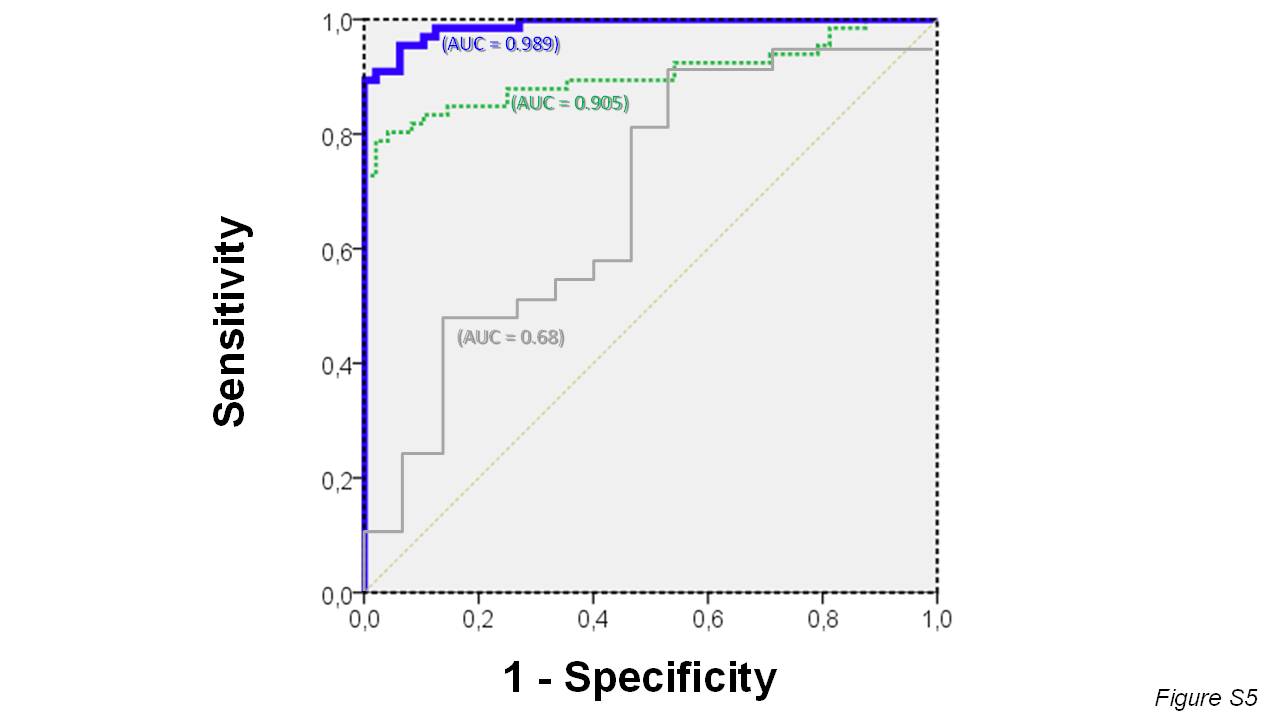

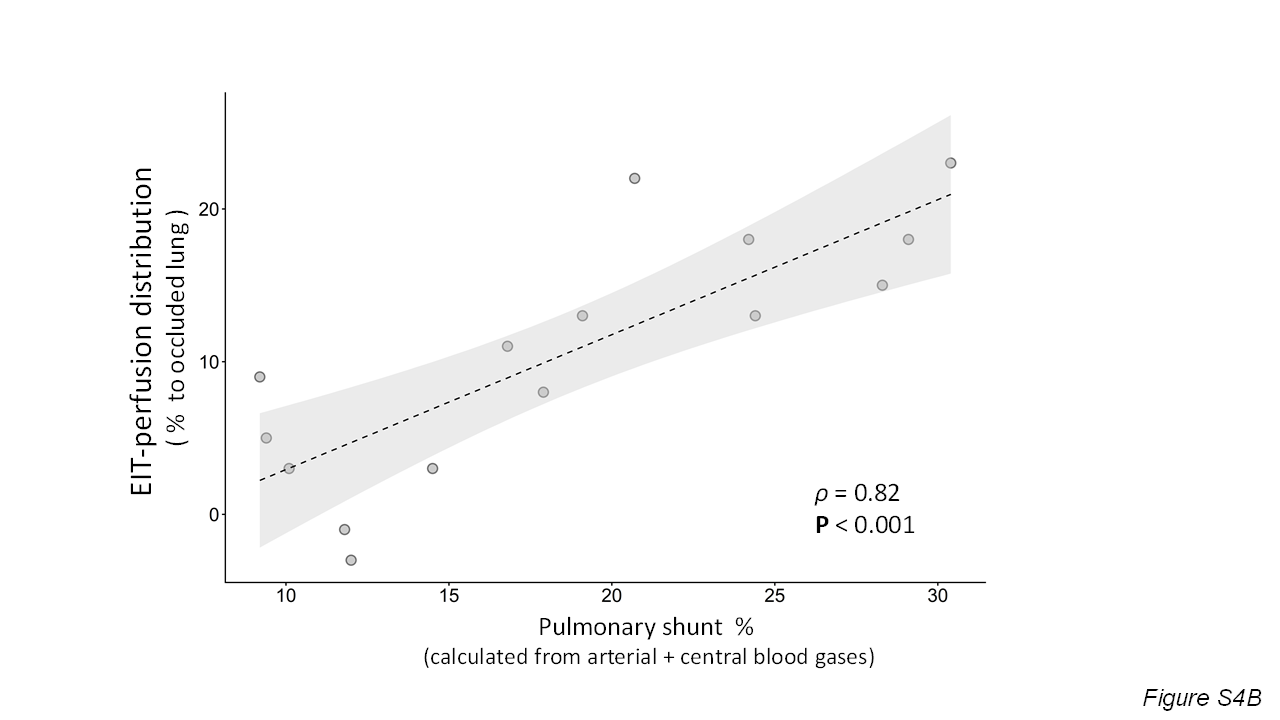

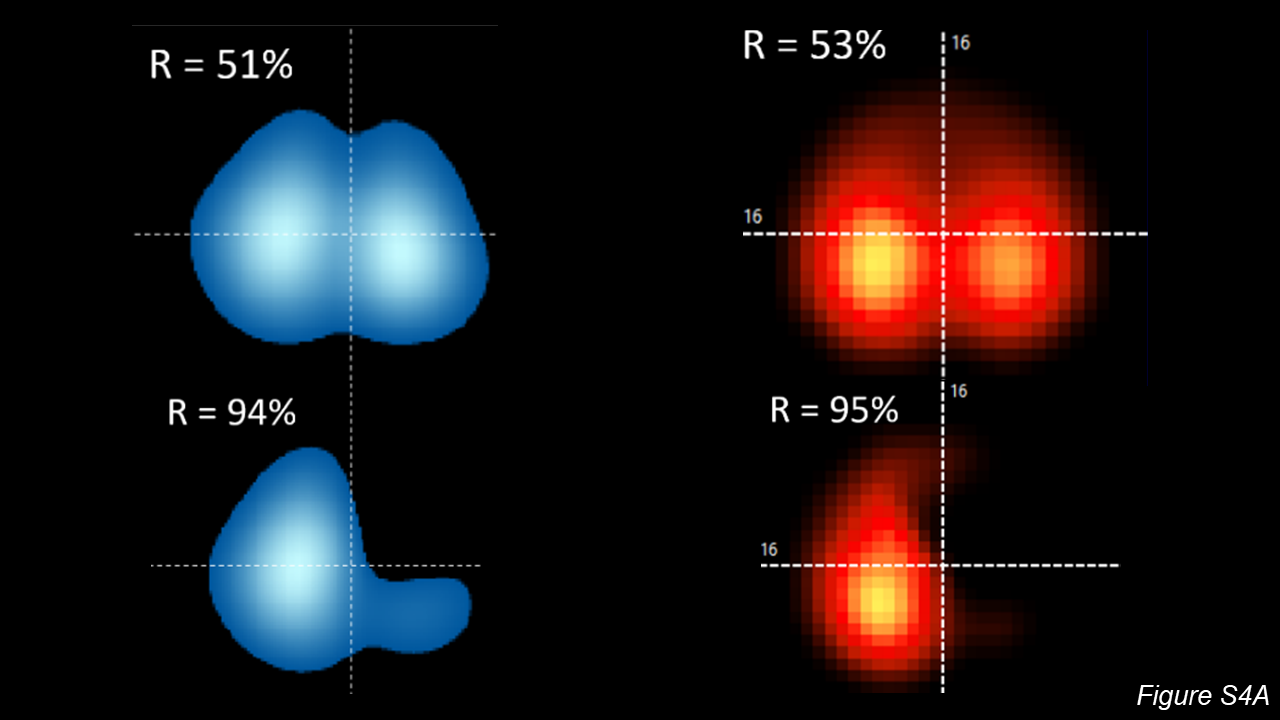

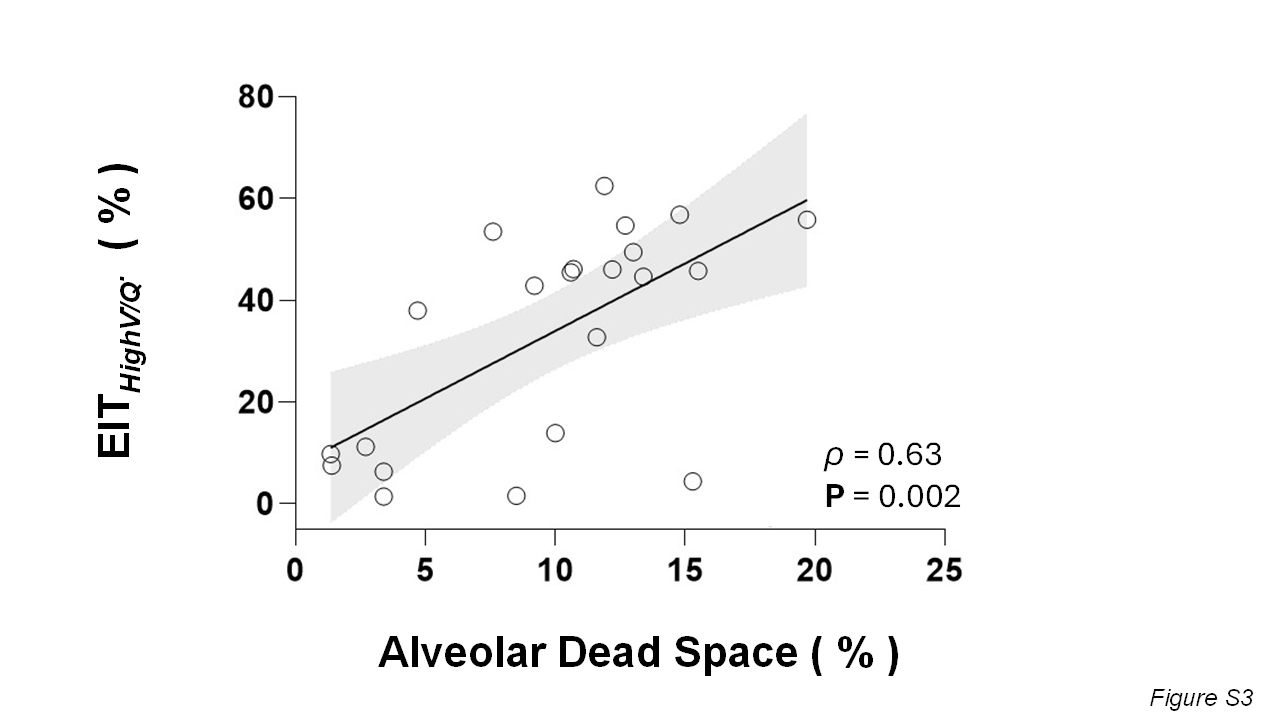

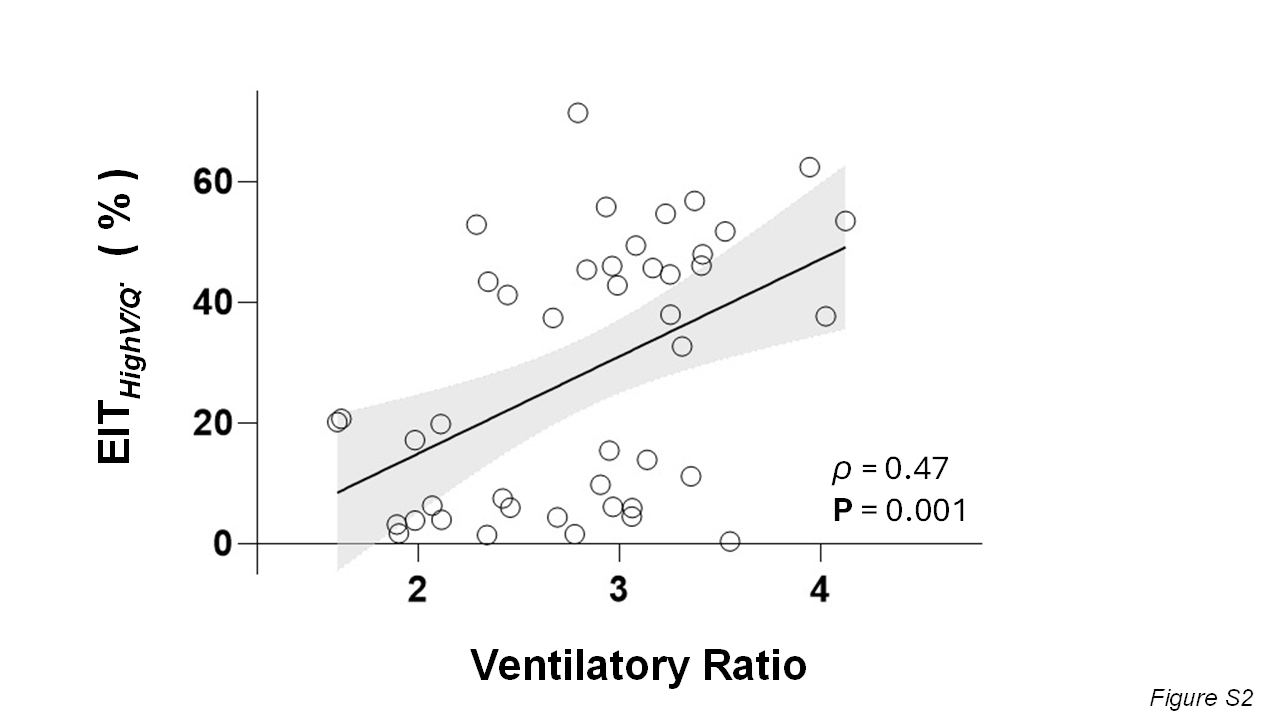

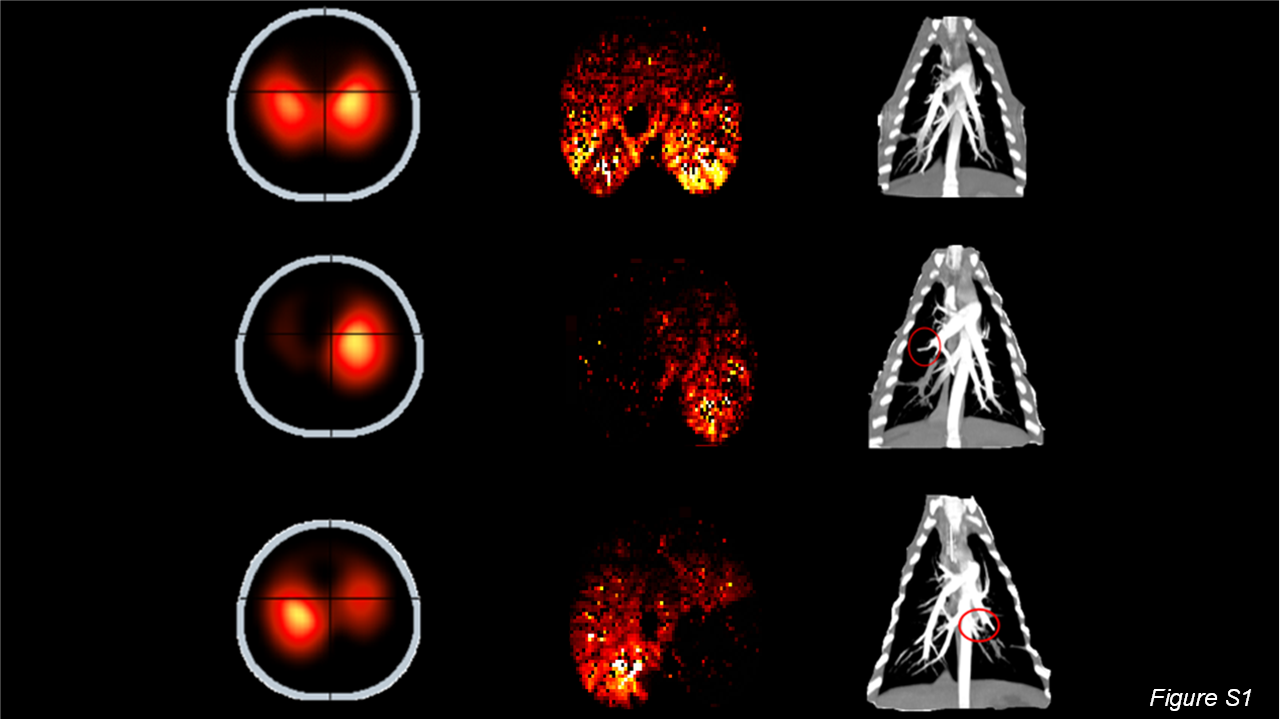

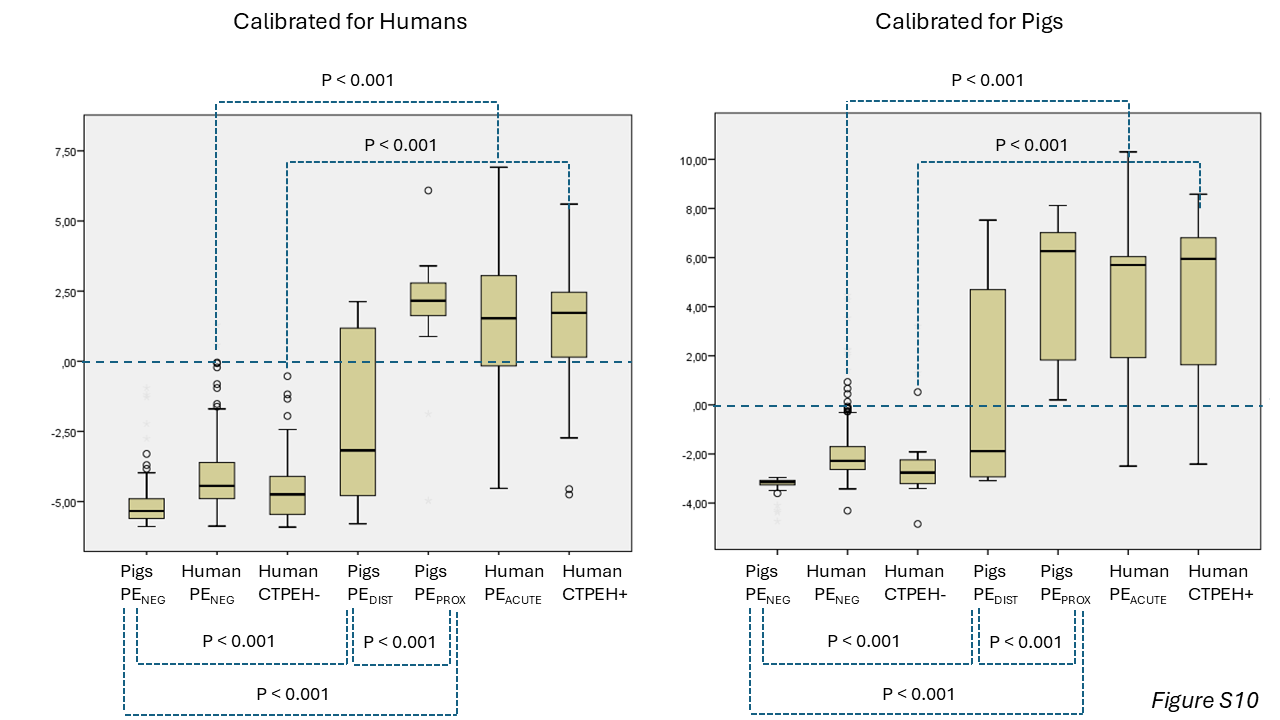


**Figure S1: Imaging studies of pulmonary perfusion distribution.**

Illustrative perfusions distributions by electrical impedance tomography (left), dynamic-contrast enhanced computed tomography (central), and computed tomography pulmonary angiography (CTPA, right), are shown. The steps baseline (upper panel), proximal pulmonary artery occlusion (in the right lung; middle panel), and distal pulmonary artery occlusion (in the left lung; bottom panel) are presented. The location of the tip of the Swan-Ganz balloon is indicated by the red circle mark within the CTPA images.

**Figure S2: Correlation between the index of *wasted-ventilation* by electrical impedance tomography and ventilatory ratio.**

We computed the index of *wasted-ventilation* by electrical impedance tomography (EIT) in the following way: the percentage of ventilation directed to pixels classified as part of the *Mostly Ventilated* EIT ventilation-perfusion (V̇/Q̇) compartment (EIT***_HighV̇/Q̇_***) within a region-of-interest (ROI), in relation to the total ventilation received by that respective ROI. Such *wasted-ventilation* index for the global ROI correlated positively with the Ventilatory Ratio: r = 0.47 (*P* = 0.001).

**Figure S3: Correlation between the index of *wasted-ventilation* by electrical impedance tomography and alveolar dead space.**

We computed the index of *wasted-ventilation* by electrical impedance tomography (EIT) in the following way: the percentage of ventilation directed to pixels classified as part of the *Mostly Ventilated* EIT ventilation-perfusion (V̇/Q̇) compartment (EIT***_HighV̇/Q̇_***) within a region-of-interest (ROI), in relation to the total ventilation received by that respective ROI. Such *wasted-ventilation* index for the global ROI correlated positive and strongly with the alveolar dead space estimated by volumetric capnography: r = 0.63, *P* = 0.002.

**Figure S4: Ventilation and perfusion maps by electrical impedance tomography and correlation with shunt during baseline and bronchial obstruction.**

A) Ventilation and perfusion maps by electrical impedance tomography (EIT) from the same animal are shown (left/blue and right/red maps, respectively). The upper row is from the step baseline (without any occlusion), and the lower one is from the bronchial obstruction step (left or right lung), creating a hypoxic environment behind the closed bronchi. The symbol R = represents the percentage of ventilation or perfusion directed to the right lung region-of-interest (ROI). Note that, despite the absence of true vascular occlusion to the left lung, perfusion showed great impairment, with 5% of cardiac output directed to the left lung. This situation configured a challenging case for our *wasted-ventilation* index, which persisted with negative values in this case.

B) Correlation between pulmonary shunt levels and EIT estimates of perfusion directed to the *target* (non-ventilated) left or right lung *ROI*: r = 0.82, (95% CI: 0.52 to 0.94), P< 0.001. Greater perfusion in non-ventilated lung regions results in higher shunt levels, as shown by simultaneous arterial and pulmonary artery blood gas sampling.

**Figure S5: Receiver operating characteristic curve analysis to assess the accuracy of ventilatory ratio in discriminating cases with and without perfusion defects.**

Plots of the receiver operating characteristic (ROC) curve for experimental data, having pulmonary embolism (PE by reference method; proximal or distal) as the dependent variable. Ventilatory-Ratio (gray, solid line) ROC curve is shown, with AUC = 0.68. Models 1 and 2 ROC curves are also shown for reference, demonstrating far greater accuracy (compared with Ventilatory-Ratio) for PE prediction.

Model 1 (green, dashed line): global index of *wasted-ventilation*, i.e. ventilation directed to EIT***_HighV̇/Q̇_*** pixels only for the global region-of-interest (ROI) variable.
Model 2 (blue, solid line): global plus local indexes of *wasted-ventilation*, i.e. ventilation directed to EIT***_HighV̇/Q̇_*** pixels for the global plus local ROIs variables.

AUC = area under the ROC curve.

**Figure S6: The response of EIT *wasted-ventilation* index to thrombolysis or pulmonary thromboendarterectomy.**

The EIT *wasted-ventilation* index was calculated according to logistic Model 2 and was previously optimized and calibrated in piglets. Subsequently it was calculated for our patients with acute pulmonary embolism (PE) or chronic thromboembolic pulmonary hypertension (CTEPH). The individual index of each patient, measured before and after thrombolysis or thrombectomy, is shown. Each color corresponds to an individual patient. Square-dashed lines = acute respiratory failure patients that were submitted to thrombolysis in the intensive care unit; circles-continuous lines = chronic thromboembolic pulmonary hypertension patients that were submitted to pulmonary thromboendarterectomy. Note that the *wasted-ventilation* index decreased in all cases, and that the cutoff of zero, chosen to correspond to the optimum Youden cutoff in piglets, seemed to work reasonably in patients. The few patients with positive values after thrombectomy may represent residual disease after surgery, suggested by residual pulmonary hypertension found in those cases.

**Figure S7: Ventilation, perfusion, and ventilation-perfusion maps by electrical impedance tomography from two representative patients.**

Electrical impedance tomography (EIT) ventilation (A), perfusion (B) and ventilation-perfusion (V̇/Q̇) maps (C) from two representative patients. The upper row is from a patient with acute hypoxemic respiratory failure but without pulmonary embolism. The lower row is from a patient with acute hypoxemic respiratory failure and confirmed pulmonary embolism (in the right lung). The corresponding indexes of *wasted*-*ventilation*, i.e. the percentage of ventilation directed to pixels classified as *Mostly Ventilated* within a region in relation to the total ventilation received by that respective region, are also shown.

Blue pixels in V̇/Q̇ map: ventilation directed to pixels classified as part of the *Mostly Ventilated* EIT V̇/Q̇ compartment (EIT***_HighV̇/Q̇_***), i.e. pixels with V̇/Q̇ > 2.0.

Green pixles in V̇/Q̇ map: ventilation directed to pixels classified as part of the *Ventilated and Perfused* EIT V̇/Q̇ compartment (EIT***_BalancedV̇/Q̇_***), i.e. pixels with V̇/Q̇ between 0.5 – 2.0.

Red pixels in V̇/Q̇ map: ventilation directed to pixels classified as part of the *Mostly Perfused* EIT V̇/Q̇ compartment (EIT***_LowV̇/Q̇_***), i.e. pixels with V̇/Q̇ < 0.5.

**Figure S8: Perfusion and ventilation-perfusion maps by electrical impedance tomography from one patient with chronic thromboembolic pulmonary hypertension.**

Electrical impedance tomography (EIT) perfusion and ventilation-perfusion (V̇/Q̇) maps from one patient with chronic thromboembolic pulmonary hypertension (CTEPH) that was submitted to a PTE (pulmonary thromboendarterectomy). The upper row shows the maps before the PTE, and the lower row shows them after that. The new *wasted-ventilation* index classified the patient correctly before (pulmonary embolism positive) and after (pulmonary embolism negative), depicting a typical CTEPH case with marked postoperative *wasted-ventilation* index reduction.

Blue pixels in V̇/Q̇ maps: ventilation directed to pixels classified as part of the *Mostly Ventilated* EIT V̇/Q̇ compartment (EIT***_HighV̇/Q̇_***), i.e. pixels with V̇/Q̇ > 2.0.

Green pixels in V̇/Q̇ maps: ventilation directed to pixels classified as part of the *Ventilated and Perfused* EIT V̇/Q̇ compartment (EIT***_BalancedV̇/Q̇_***), i.e. pixels with V̇/Q̇ between 0.5 – 2.0.

Red pixels in V̇/Q̇ maps: ventilation directed to pixels classified as part of the *Mostly Perfused* EIT V̇/Q̇ compartment (EIT***_LowV̇/Q̇_***), i.e. pixels with V̇/Q̇ < 0.5.

**Figure S9: Perfusion and ventilation-perfusion maps by EIT from two patients with chronic thromboembolic pulmonary hypertension.**

Electrical impedance tomography (EIT) perfusion and ventilation-perfusion (V̇/Q̇) maps from two patients with chronic thromboembolic pulmonary hypertension (CTEPH) that were submitted to a PTE (pulmonary thromboendarterectomy). In both clinical cases (S9A and S9B), upper row shows the maps before PTE, and the lower row shows them after PTE. In both cases, the new *wasted-ventilation* index classified the patients correctly before PTE (pulmonary embolism positive). In the first case, the new *wasted-ventilation* index also classified the patient correctly after PTE (pulmonary embolism negative). And both cases behaved as typical CTEPH cases with marked postoperative index reductions. In the second case, the slightly positive index after surgery was suggestive of residual disease after the procedure, suggested by the particularly high degree of hypertension and right ventricular disfunction after the procedure.

Blue pixels: ventilation directed to pixels classified as part of the *Mostly Ventilated* EIT V̇/Q̇ compartment (EIT***_HighV̇/Q̇_***), i.e. pixels with V̇/Q̇ > 2.0.

Green pixels: ventilation directed to pixels classified as part of the *Ventilated and Perfused* EIT V̇/Q̇ compartment (EIT***_BalancedV̇/Q̇_***), i.e. pixels with V̇/Q̇ between 0.5 – 2.0.

Red pixels: ventilation directed to pixels classified as part of the *Mostly Perfused* EIT V̇/Q̇ compartment (EIT***_LowV̇/Q̇_***), i.e. pixels with V̇/Q̇ < 0.5.

**Figure S10: Discrimination between positive and negative pulmonary embolism cases.**

Two calibrations for Model 2 are presented, one specific for humans and another specific for piglets. The data demonstrates that the discrimination between positive and negative pulmonary embolism (PE) cases, when considering piglets and humans separately, is remarkable. Also, the similarity between human and piglet cases is also remarkable, independently of the calibration. As expected, proximal PE in piglets were much better discriminated against negative cases than in distal occlusion cases. Indeed, the difference of scores between proximal and distal PE is very significant (*P* < 0.001). The two calibrations produced similar Models with the same rationale and with the same emphasis on the good predictive power of dorsal quadrants.

PE_NEG_: negative for pulmonary embolism.

CTEPH-: negative for pulmonary embolism in patients with chronic thromboembolic pulmonary hypertension.

PE_DISTAL_: positive for distal pulmonary embolism.

PE_PROX_: positive for proximal pulmonary embolism.

PE_ACUTE_: positive for acute pulmonary embolism.

CTEPH+: positive for pulmonary embolism in patients with chronic thromboembolic pulmonary hypertension.

**Tables**

**Table S1: Individual Baseline Characteristics of the Animals of the Experimental Model of Pulmonary Embolism.**

*Definition of abbreviations*: PEEP = positive end-expiratory pressure; C_rs_ = respiratory system compliance.

**Table S2: Acute Hypoxemic Respiratory Failure Patient Characteristics.**

*Definition of abbreviations*: ABW = actual body weight; PBW = predicted body weight; BMI = body mass index; PEEP = positive end-expiratory pressure at study baseline; PaO_2_/F_I_O_2_ = the ratio of arterial partial pressure of oxygen (PaO_2_) to the fraction of inspired oxygen (F_I_O_2_) at study baseline; SAPS II = Simplified Acute Physiology Score II.

^a^Data within brackets are presented as median [interquartile range].

**Table S3A: Chronic Thromboembolic Pulmonary Hypertension Patient Characteristics.**

*Definition of abbreviations*: Pts = patients; F = female; M = male; ABW = actual body weight; PBW = predicted body weight; BMI = body mass index; CTEPH: chronic thromboembolic pulmonary hypertension; UCSD^a^ = University of California San Diego surgical classification^a^; *R* = right lung; *L* = left lung; SAPS III = Simplified Acute Physiology Score III. ^a^This is a new surgical classification that has been developed to reflect the level of disease (i.e., lobar, segmental, subsegmental) as opposed to the type of disease [11, 17, 18]. It is based on the most proximal disease identified in each pulmonary artery, and right and left. It is done by the surgeon, after removing the thrombi, and it stratifies it into the following levels: level 0, no evidence of chronic thromboembolic (CTE) disease; level 1, CTE disease encountered in the main pulmonary artery; level 2, CTE disease starting at the level of lobar arteries, or in the main descending pulmonary arteries; level 3, CTE disease starting at the level of the segmental arteries; level 4, CTE disease starting at the level of the subsegmental arteries. Levels 1-3 lesions are operable with pulmonary thromboendarterectomy (PTE), while level 4 lesions are usually inoperable.

**Table S3B: Mechanical Ventilation Settings and Respiratory Mechanics of the Chronic Thromboembolic Pulmonary Hypertension Patients.**

*Definition of abbreviations*: V_T_ = tidal volume; VE = minute volume; PEEP = positive end-expiratory pressure; P_plat_ = plateau pressure; DP = driving pressure; C_rs_ = respiratory system compliance; PaO_2_/F_I_O_2_ = the ratio of arterial partial pressure of oxygen (PaO_2_) to the fraction of inspired oxygen (F_I_O_2_); OR = at operating room; ICU^a^ = at Intensive Care Unit (ICU) admission.

**Table S3C: Hemodynamic Data of the Chronic Thromboembolic Pulmonary Hypertension Patients.**

*Definition of abbreviations*: PAP_systolic_ = systolic pulmonary arterial pressure; PAP_diastolic_ = diastolic pulmonary arterial pressure; PAP_mean_ = mean pulmonary arterial pressure; PCWP = pulmonary capillary wedge pressure; CI = cardiac index; SV = stroke volume; SVI = stroke volume index; PVR = pulmonary vascular resistance; PVRI = pulmonary vascular resistance index.

**Table S4: Best Elected Logistic Regression Model to Predict Vascular Occlusion for the Experimental Model of Pulmonary Embolism.**

| **Electrical Impedance Tomography** | **B** | **S.E.** | **Sig.** | **Exp (B)** |
| --- | --- | --- | --- | --- |
| ***Region-of-Interest (ROI)*** |  |  |  |  |
|  |  |  |  |  |
| **Global** | **4.99** | **1.77** | **0.005** | **147.24** |
| **Right Lung** | **-2.89** | **1.03** | **0.005** | **0.06** |
| **Left Lung** | **-2.32** | **0.82** | **0.005** | **0.10** |
| **Lower Right Quadrant** | **3.35** | **1.20** | **0.005** | **28.56** |
| **Lower Left Quadrant** | **1.24** | **0.52** | **0.018** | **3.47** |
| **Constant** | **-5.97** | **2.13** | **0.005** | **0.00** |
|  |  |  |  |  |

*Definition of abbreviations*: *B* = unstandardized regression coefficient; S.E. = standard error of the coefficient; Sig. = statistical significance (*P* value); Exp (B) = exponentiated coefficient, which quantifies how the odds of the outcome change when the predictor variable increases by one unit, holding all other variables constant.

**Table S5: Bootstrapping Resampling Analysis.**

Note the similarity between the coefficients *B* of Table S4 (conventional multivariate logistic regression) and the coefficients *B* of this Table S5 (obtained after averaging 1000 regressions with bootstrapping resampling of our piglet population).

*Definition of abbreviations*: *B* = unstandardized regression coefficient; C.I. = confidence interval.

**Table S6: Logistic Regression Model to Predict Vascular Occlusion - Calibrated in Humans and Later Applied to Humans.**

| **Electrical Impedance Tomography** | ***B*** | **S.E.** | **Sig.** | **Exp (B)** |
| --- | --- | --- | --- | --- |
| ***Region-of-Interest (ROI)*** |  |  |  |  |
|  |  |  |  |  |
| **Global** | **0.11** | **0.05** | **0.011** | **1.12** |
| **Right Lung** | **-0.06** | **0.04** | **0.133** | **0.94** |
| **Left Lung** | ****** | **---** | **N.S.** | **---** |
| **Lower Right Quadrant** | **0.08** | **0.02** | **<0.001** | **1.08** |
| **Lower Left Quadrant** | **0.03** | **0.04** | **0.051** | **1.03** |
| **Constant** | **-5.89** | **0.90** | **0.003** | **0.003** |
|  |  |  |  |  |

*Definition of abbreviations*: *B* = unstandardized regression coefficient; S.E. = standard error of the coefficient; Sig. = statistical significance (*P* value); Exp (B) = exponentiated coefficient, which quantifies how the odds of the outcome change when the predictor variable increases by one unit, holding all other variables constant.

****** We removed the “Left Lung” variable from the regression equation for two reasons: it was not significant (*P* > 0.50), and because its presence was causing spurious results in the coefficients of the Lower Left Quadrant.

**References**

1. Ribeiro BM, Tucci MR, Victor Junior MH, Melo JR, Gomes S, Nakamura MAM, Morais CCA, Beraldo MA, Lima CAS, Alcala GC *et al*: **Influence of Fractional Inspired Oxygen Tension on Lung Perfusion Distribution, Regional Ventilation, and Lung Volume during Mechanical Ventilation of Supine Healthy Swine**. *Anesthesiology* 2024, **140**(4):752-764.

2. Costa EL, Borges JB, Melo A, Suarez-Sipmann F, Toufen C, Jr., Bohm SH, Amato MB: **Bedside estimation of recruitable alveolar collapse and hyperdistension by electrical impedance tomography**. *Intensive Care Med* 2009, **35**(6):1132-1137.

3. Victorino JA, Borges JB, Okamoto VN, Matos GF, Tucci MR, Caramez MP, Tanaka H, Sipmann FS, Santos DC, Barbas CS *et al*: **Imbalances in regional lung ventilation: a validation study on electrical impedance tomography**. *Am J Respir Crit Care Med* 2004, **169**(7):791-800.

4. Frerichs I: **Electrical impedance tomography (EIT) in applications related to lung and ventilation: a review of experimental and clinical activities**. *Physiol Meas* 2000, **21**(2):R1-21.

5. Borges JB, Suarez-Sipmann F, Bohm SH, Tusman G, Melo A, Maripuu E, Sandstrom M, Park M, Costa EL, Hedenstierna G *et al*: **Regional lung perfusion estimated by electrical impedance tomography in a piglet model of lung collapse**. *J Appl Physiol (1985)* 2012, **112**(1):225-236.

6. Xin Y, Kim T, Winkler T, Brix G, Gaulton T, Gerard SE, Herrmann J, Martin KT, Victor M, Reutlinger K *et al*: **Improving pulmonary perfusion assessment by dynamic contrast-enhanced computed tomography in an experimental lung injury model**. *J Appl Physiol (1985)* 2023, **134**(6):1496-1507.

7. Brix G, Zwick S, Griebel J, Fink C, Kiessling F: **Estimation of tissue perfusion by dynamic contrast-enhanced imaging: simulation-based evaluation of the steepest slope method**. *Eur Radiol* 2010, **20**(9):2166-2175.

8. Jonkman AH, Alcala GC, Pavlovsky B, Roca O, Spadaro S, Scaramuzzo G, Chen L, Dianti J, Sousa MLA, Sklar MC *et al*: **Lung Recruitment Assessed by Electrical Impedance Tomography (RECRUIT): A Multicenter Study of COVID-19 Acute Respiratory Distress Syndrome**. *Am J Respir Crit Care Med* 2023, **208**(1):25-38.

9. Brookes JDL, Li C, Chung STW, Brookes EM, Williams ML, McNamara N, Martin-Suarez S, Loforte A: **Pulmonary thromboendarterectomy for chronic thromboembolic pulmonary hypertension: a systematic review**. *Ann Cardiothorac Surg* 2022, **11**(2):68-81.

10. Madani MM: **Surgical Treatment of Chronic Thromboembolic Pulmonary Hypertension: Pulmonary Thromboendarterectomy**. *Methodist Debakey Cardiovasc J* 2016, **12**(4):213-218.

11. Delcroix M, Torbicki A, Gopalan D, Sitbon O, Klok FA, Lang I, Jenkins D, Kim NH, Humbert M, Jais X *et al*: **ERS statement on chronic thromboembolic pulmonary hypertension**. *Eur Respir J* 2021, **57**(6).

12. Kim NH, Delcroix M, Jais X, Madani MM, Matsubara H, Mayer E, Ogo T, Tapson VF, Ghofrani HA, Jenkins DP: **Chronic thromboembolic pulmonary hypertension**. *Eur Respir J* 2019, **53**(1).

13. Madani MM, Wiedenroth CB, Jenkins DP, Fadel E, de Perrot M: **Pulmonary Thromboendarterectomy: The Potentially Curative Treatment of Choice for Chronic Thromboembolic Pulmonary Hypertension**. *Ann Thorac Surg* 2025, **119**(4):756-767.

14. Fumagalli J, Santiago RRS, Teggia Droghi M, Zhang C, Fintelmann FJ, Troschel FM, Morais CCA, Amato MBP, Kacmarek RM, Berra L *et al*: **Lung Recruitment in Obese Patients with Acute Respiratory Distress Syndrome**. *Anesthesiology* 2019, **130**(5):791-803.

15. Kline JA, Courtney DM, Kabrhel C, Moore CL, Smithline HA, Plewa MC, Richman PB, O'Neil BJ, Nordenholz K: **Prospective multicenter evaluation of the pulmonary embolism rule-out criteria**. *J Thromb Haemost* 2008, **6**(5):772-780.

16. Kahn SR, de Wit K: **Pulmonary Embolism**. *N Engl J Med* 2022, **387**(1):45-57.

17. Fernandes TM, Kim NH, Kerr KM, Auger WR, Fedullo PF, Poch DS, Yang J, Papamatheakis DG, Alotaibi M, Bautista MA *et al*: **Distal vessel pulmonary thromboendarterectomy: Results from a single institution**. *J Heart Lung Transplant* 2023, **42**(8):1112-1119.

18. de Perrot M, Gopalan D, Jenkins D, Lang IM, Fadel E, Delcroix M, Benza R, Heresi GA, Kanwar M, Granton JT *et al*: **Evaluation and management of patients with chronic thromboembolic pulmonary hypertension - consensus statement from the ISHLT**. *J Heart Lung Transplant* 2021, **40**(11):1301-1326.
